# Exploring structure–activity relationships of pyrrolyl diketo acid derivatives as non-nucleoside inhibitors of terminal deoxynucleotidyl transferase enzyme

**DOI:** 10.1080/14756366.2025.2496782

**Published:** 2025-06-09

**Authors:** Valentina Noemi Madia, Nadia Garibaldi, Davide Ialongo, Elisa Patacchini, Valeria Tudino, Giuseppe Ruggieri, Laura Zarbo, Emanuele Cara, Antonio Coluccia, Marco Artico, Luigi Scipione, Antonella Messore, Francesco Saccoliti, Elisa Mentegari, Giovanni Maga, Roberto Di Santo, Emmanuele Crespan, Roberta Costi

**Affiliations:** ^a^ Istituto Pasteur-Fondazione Cenci Bolognetti, Dipartimento di Chimica e Tecnologie del Farmaco, “Sapienza” Università di Roma, Rome, Italy; ^b^ Institute of Molecular Genetics IGM-CNR, Pavia, Italy; ^c^ Department of Biotechnology, Chemistry and Pharmacy, University of Siena, Siena, Italy; ^d^ Dottorato di Interesse Nazionale in One Health approaches to infectious diseases and life science research, Dipartimento di Sanità Pubblica, Medicina Sperimentale e Forense, Università degli Studi di Pavia, Pavia, Italy; ^e^ Department of Sense Organs, Faculty of Medicine and Odontology, “Sapienza” Università di Roma, Rome, Italy; ^f^ Department of Life Science, Health, and Health Professions, Link Campus University, Rome, Italy

**Keywords:** DNA repair, terminal deoxynucleotidyl transferase, medicinal chemistry, drug discovery, structure–activity relationships

## Abstract

Terminal deoxynucleotidyl transferase (TdT) is overexpressed in some cancer types, where it drives the mutagenic repair of double strand breaks through non canonical non-homologous end joining pathway. The TdT enzyme belongs to the X family of polymerases, together with the DNA polymerase λ (pol λ) and β (pol β). However, TdT exclusively displays template-independent nucleotide polymerisation. Pursuing our studies in developing TdT inhibitors, herein we deepened the structure-activity relationships of new structural analogues of our previously identified hit compounds. The diketo hexenoic acid derivatives here analysed showed high selectivity towards TdT and inhibition potencies spanning from the low micromolar range to the nanomolar. Docking studies highlighted the chemical features involved in the TdT binding, well contributing to the rationalisation of the structural requirements needed for the enzymatic inhibition.

## Graphical Abstract

**Figure:**
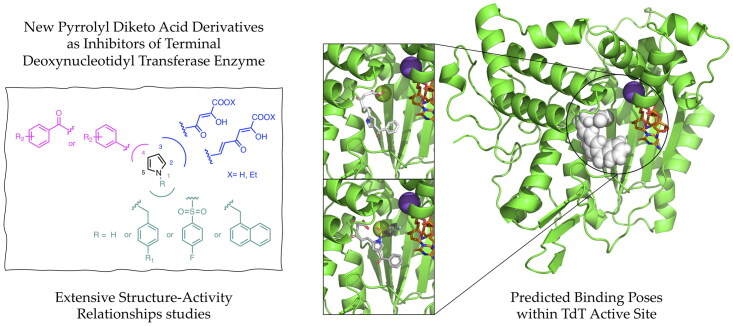

## Introduction

Differently from canonical DNA polymerases, the terminal deoxynucleotidyl transferase (TdT) catalyses DNA polymerisation without requiring a template strand but only adding deoxynucleotides to the 3′ hydroxyl terminus of single-stranded DNA strand^1^^,^^2^. TdT belongs to the X family of polymerases, together with the DNA polymerase lambda (pol λ) and polymerase beta (pol β). However, while TdT exclusively displays template-independent nucleotide polymerisation, pol λ is considered a “hybrid” enzyme, possessing both canonical template-dependent (DNA polymerase) and template-independent (terminal transferase) activities. Considering that the common ancestor of X family members was a template-dependent polymerase^3^, the TdT terminal transferase activity can be viewed as a relatively recent evolutionary acquisition. This evolutionary perspective is consistent with the pivotal role of TdT in V(D)J recombination. During the development of B and T cells in the bone marrow and thymus, this complex series of events leads to the generation of the exceptional diversity within the antigen-binding region of immunoglobulins (Ig) and T-cell receptors^4^. TdT takes part in this pathway catalysing the non-germline-encoded terminal nucleotide (N-) addition at the junctions of V(D)J gene segments within Ig and T-cell receptor loci. Such random incorporation of nucleotides contributes to unique antigen receptor sequences^5^^,^^6^. This activity is finely regulated. Downregulation of TdT in mice results in suppression of (N-) addition and its expression is precisely tuned in time, occurring prior to D–J rearrangement and ceasing during Ig expression. TdT is not present during foetal and neonatal life, with its expression in these life stages impairing the ability to produce protective antibodies in mice^7^.

Soon after its discovery, TdT was found overexpressed in leukaemias^8–10^. TdT is expressed in 90% of the blast cells of B- and T-cell acute lymphocytic leukaemias (ALLs)^9^, showing a large variability in expression levels – up to 1000-fold variation – and multiple isoforms patterns among individual tumours. The same is true for 18% of acute myelocytic leukaemias (AML) and 30% of chronic myelocytic leukaemia (CML). However, different studies attribute different prognostic values to TdT expression^11–13^.

Despite decades passed from the discovery of TdT overexpression in lymphoid tumours, its pathophysiology role has not been extensively investigated yet. In ALL B-cells, robust evidence describes abnormal rearrangements in T-cell receptors (TCRs) and the same is true in AML also including B-cell receptor (BCR) genes, which includes long nucleotide additions and deletions of V and J coding regions.

These observations suggest that aberrant expression and activity of TdT may exert a mutagenic effect, enabling genome instability in cancer perturbing canonical double-strand breaks repair (DSBs). This suggestion is fostered by the observation that the expression of TdT in U2OS cells suppresses faithful DSBs repair based on short homology regions, while promoting addition of anonymous nucleotides^14^. Moreover, ectopic expression of TdT in CHO cells promotes N-addition at non-V(D)J DSBs, through a process that requires the components of the canonical non-homologous end-joining pathway^15^.

Other than leukaemias, TdT has been found highly expressed in the majority of Merkel cell carcinomas, a rare form of skin cancer characterised by rapid progression of malignancy^16^. More recently, TdT has been found also expressed in germ cell tumors^16–18^. The contribution of TdT in these cancers has not been explored, yet, but given its role in leukaemias, it has been proposed as a possible target for cancer therapy. Indeed, TdT splicing modulation and downregulation results in induction of apoptosis and reduced cell survival in Molt-4 cells^13^^,^^19^. In T-acute lymphoblastic leukaemia/lymphoma (T-ALL/LBL), TdT knockout delayed DNA repair, arrested the cell cycle and induced the accumulation of chromosomal abnormalities^13^.

**Figure 1.**
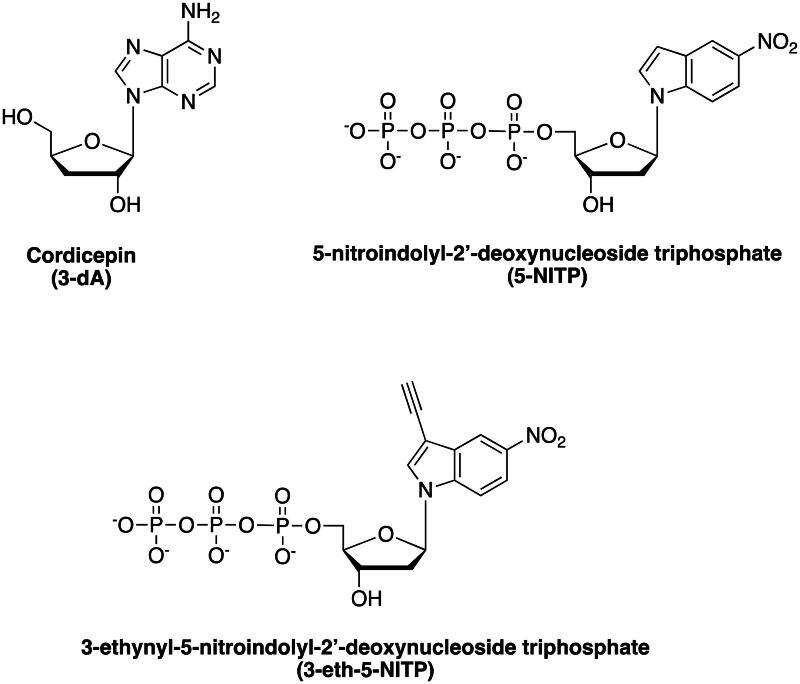
Structures of the nucleotidyl analogues cordycepin (3-dA), 5-nitroindolyl-2’-deoxynucleoside triphosphate (5-NITP), and 3-ethynyl-5-nitroindolyl-2′-deoxynucleoside triphosphate (3-eth-5-NITP).

Different approaches to develop effective inhibitors of TdT have been attempted. Nucleoside analogues acting as chain terminators constitute a first class of TdT inhibitors. Cordycepin (3-dA) belongs to this class of inhibitors (Figure 1). In the presence of the antitumor agent deoxycoformycin, 3-dA demonstrated selective cytotoxicity against TdT-positive cells *in vitro*^20^^,^^21^. Indeed, it has been reported that 3-dA showed IC_50_ of 0.92 μM in MOLT-4 cells in the presence of 2.5 μM deoxycoformycin^21^. However, in contrast to these preclinical studies, 3-dA failed to exhibit anti-tumor activity in clinical trials^22^. Notably, 3-dA is capable of interfering with purine synthesis and mTOR signal transduction that, together with its low selectivity against other cellular DNA polymerases, contributes to increased adverse effects^23^. To address this challenge, Motea *et al* developed the nucleoside analogue 5-nitroindolyl-2′-deoxynucleoside triphosphate (5-NITP, Figure 1) that is selectively utilised by TdT resulting in cytostatic and cytotoxic effects against leukaemia cells overexpressing TdT^24^^,^^25^. Importantly, the ethynyl moiety in 5-NITP (3-eth-5-NITP, Figure 1) allows the efficient and selective labelling of the incorporated non-natural nucleotide with an azide-containing fluorophore through click chemistry. This enables the quantitative assessment of nucleotide incorporation, demonstrating that the anticancer effects of 3-eth-5-NITP correlate in a dose-dependent manner to TdT levels. Nonetheless, in biochemical assays, 3-eth-5-NITP exhibits potency of action in the micromolar range, reflecting moderate cytotoxicity activity (IC_50_ of 3.90 μM)^24^.

**Figure 2.**
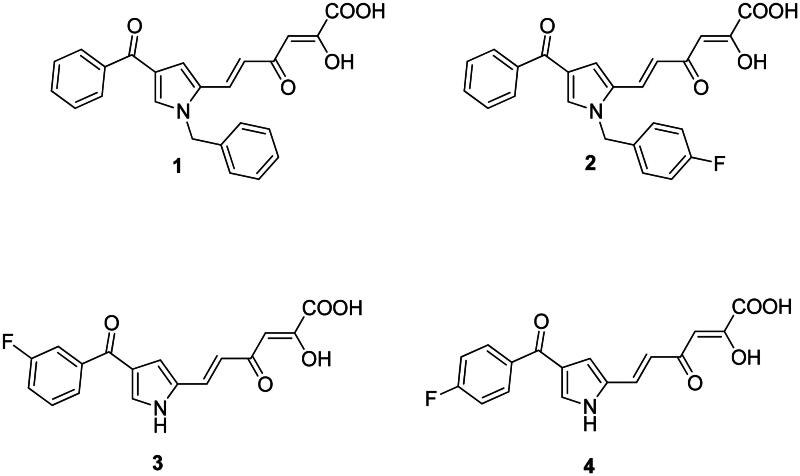
Previously identified non-nucleoside TdT inhibitors **1**–**4**.

Moreover, nucleoside analogues exert higher severity of side effects than non-nucleoside ones by mimicking endogenous nucleosides (and following phosphorylation, nucleotides). Therefore, taking into account all aforementioned, we devoted our efforts in the development of new non-nucleoside analogues as inhibitors of TdT, identifying a group of aryl diketo hexenoic acid (DKA) compounds as potent and selective inhibitors of TdT. Among them, compounds **1** and **2** (Figure 2, Tables 1 and 2) showed a strong cytotoxic effect against the TdT-positive leukaemia cell line MOLT-4, compared to the TdT-negative cell lines derived from cervical cancer, HeLa^26–28^. Later, we reported a series of diketo hexenoic analogues as the first non-nucleoside TdT inhibitors endowed with selective activity. Among these newly identified TdT inhibitors, the most potent compound **3** and **4** (Figure 2, Tables 1 and 2) were cocrystallised with TdT at a resolution of 2.4 − 2.6 Å. The structures of the complexes between the nucleotide-competitive inhibitors **3** and **4** and TdT showed that these compounds physically interfere with the binding of the incoming nucleotide. Both are bound near to the active site of TdT^28^.

**Figure 3.**
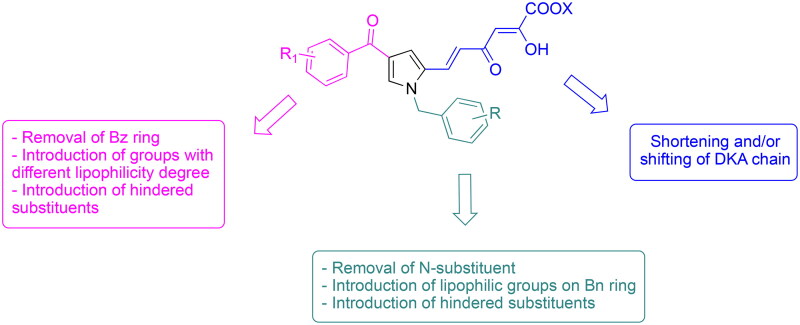
Design of the newly synthesised TdT inhibitors.

**Table 1.** Inhibitory activities of newly synthesised diketo hexenoic derivatives **5a**-**m**, **6a**-**m**, **7a**-**d**, **8a**-**d** and diketo butanoic derivatives **9a**,**b**, **10a**,**b**, **11a**,**b** and **12a**,**b** against human TdT.

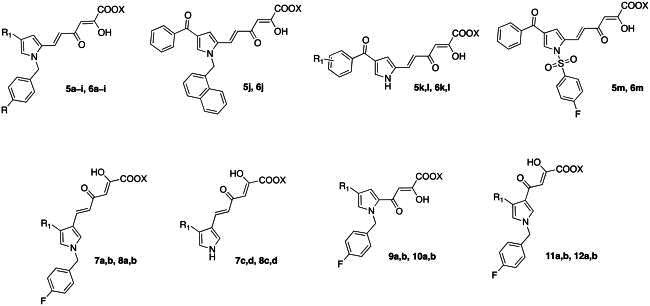					
Cpd	R	R_1_	X	ID_50_ (µM)^*a*^	
				Mg^2+^	Mn^2+^
**5a**	F	H	Et	nd^*b*^	>50
**5b**	F	Ph^*c*^	Et	nd	>50
**5c**	F	2,3-dihydrobenzo[*b*][1,4]dioxine-6-carbonyl	Et	nd	>50
**5d**	F	4-(naphthalen-2-yl-OCH_3_)Bz	Et	5.83 ± 1.76	>50
**5e**	F	4-phenoxy-Bz	Et	nd	>50
**5f**	F	4-NO_2_-Bz	Et	6.54 ± 0.78	>50
**5g**	F	4-(3-Br-4-OCH_3_-benzamido)Bz	Et	0.91 ± 0.36	>50
**5h**	Cl	Bz^*d*^	Et	17.51 ± 0.12	>50
**5i**	OCH_3_	Bz	Et	10.54 ± 0.20	>50
**5j**	–	–	Et	3.68 ± 1.00	>50
**5k**	–	3,4,5-trimethoxy-Bz	Et	10.61 ± 3.6	1.28 ± 0.27
**5l**	–	H	Et	35.82 ± 5.38	>50
**5m**	–	–	Et	3.87 ± 0.55	8.75 ± 0.35
**6a**	F	H	H	>50	1.63 ± 0.35
**6b**	F	Ph	H	>50	6.90 ± 2.5
**6c**	F	2,3-dihydrobenzo[*b*][1,4]dioxine-6-carbonyl	H	nd	4.07 ± 0.81
**6d**	F	4-(naphthalen-2-yl-OCH_3_)Bz	H	12.76 ± 4.2	>50
**6e**	F	4-phenoxy-Bz	H	nd	>50
**6f**	F	4-NO_2_-Bz	H	0.64 ± 0.25	7.71 ± 2.4
**6g**	F	4-(3-Br-4-OCH_3_-benzamido)Bz	H	0.41 ± 0.03	>50
**6h**	Cl	Bz	H	5.10 ± 0.12	nd
**6i**	OCH_3_	Bz	H	2.70 ± 0.32	>50
**6j**	–	–	H	2.11 ± 0.50	>50
**6k**	–	3,4,5-trimethoxy-Bz	H	0.86 ± 0.14	0.38 ± 0.06
**6l**	–	H	H	0.22 ± 0.01	0.30 ± 0.13
**6m**	–	–	H	0.34 ± 0.01	0.33 ± 0.05
**7a**	–	H	Et	nd	>50
**7b**	–	Ph	Et	nd	2.72 ± 0.73
**7c**	–	Ph	Et	10.5 ± 3.6	>50
**7d**	–	3,4,5-trimethoxyphenyl	Et	21.29 ± 5.05	>50
**8a**	–	H	H	nd	6.31 ± 2.8
**8b**	–	Ph	H	nd	0.66 ± 0.12
**8c**	–	Ph	H	0.11 ± 0.03	0.19 ± 0.08
**8d**	–	3,4,5-trimethoxyphenyl	H	0.24 ± 0.03	0.23 ± 0.03
**9a**	–	H	Et	nd	>50
**9b**	–	Ph	Et	nd	>50
**10a**	–	H	H	nd	1.16 ± 0.35
**10b**	–	Ph	H	nd	3.25 ± 0.66
**11a**	–	H	Et	nd	>50
**11b**	–	Ph	Et	nd	>50
**12a**	–	H	H	nd	2.80 ± 0.7
**12b**	–	Ph	H	nd	10.10 ± 4.1
**1**	–	–	–	nd	16.0 ± 0.5
**2**	–	–	–	nd	9.50 ± 0.1
**3**	–	–	–	nd	0.78 ± 0.04
**4**	–	–	–	nd	0.58 ± 0.03

^a^ Inhibitory concentration 50% (μM) determined from dose − response curves in presence of Mg^2^**^+^** or Mn^2+^.

^b^ nd: not determined.

^c^ Ph: Phenyl.

^d^ Bz: Benzoyl.

**Table 2.** Inhibitory activities of compounds **5h,i**, **6h,i** against pol **λ**.

	enzymatic assay, ID_50_ (µM)^*a*^		
Cpd	pol λ (Pol)	pol λ (TdT)	pol β
**5h**	4.08 ± 0.4	> 100	12.36 ± 0.3
**5i**	4.07 ± 0.3	> 100	7.94 ± 0.1
**6h**	4.06 ± 0.2	> 100	19.95 ± 0.4
**6i**	4.06 ± 0.1	> 100	14.67 ± 0.8
**1** ^*b*^	> 40	20 ± 1	> 40
**2** ^*b*^	> 40	> 40	> 40
**3** ^*b*^	> 40	35 ± 1	> 40
**4** ^*b*^	> 40	40 ± 1	> 40

^a^ Inhibitory concentration 50% (μM) determined from dose − response curves.

^b^ Data from reference^30^.

In this work, we describe a new series of DKA derivatives structurally related to the previous identified compounds to deepen the structure-activity relationships within this class of compounds. More in detail, the benzyl ring in position 1 of the pyrrole core has been removed, or replaced with more lipophilic or hindered substituents. A similar approach has been applied to the benzoyl group in position 4 of the central core, that has been removed or replaced with groups characterised by different degrees of steric hindrance and of hydrophilicity/lipophilicity. Finally, the diketo hexenoic chain has been shortened into a diketo butanoic one and/or shifted from position 2 to 3 (Figure 3).

**Figure 4.**
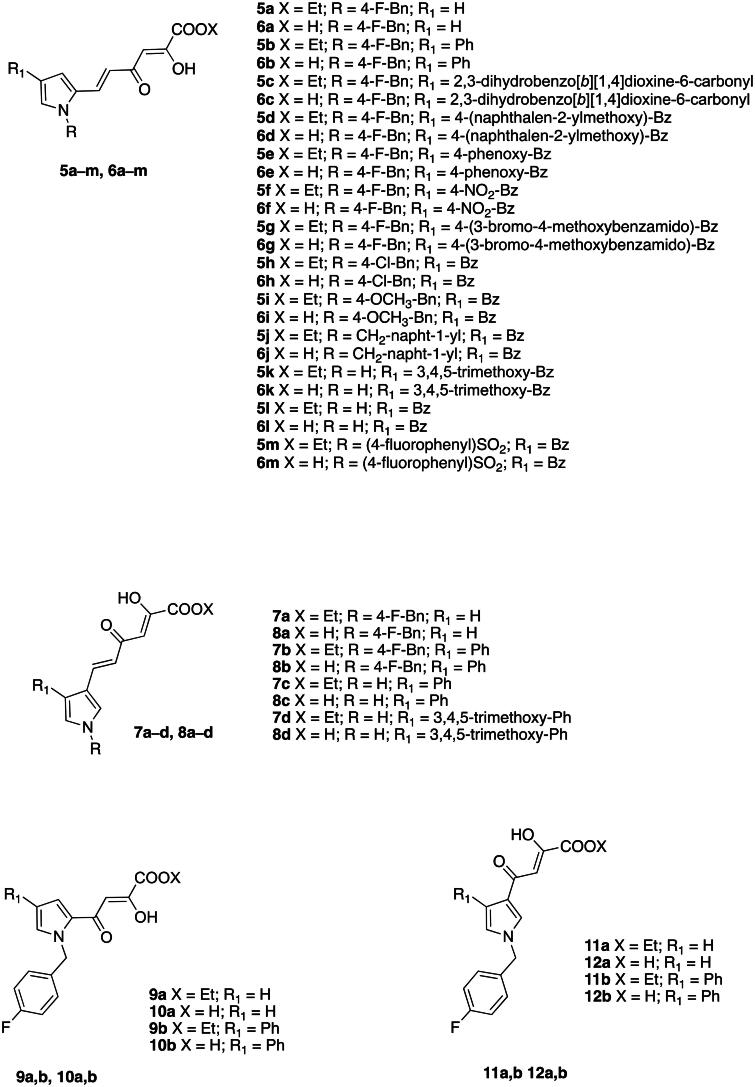
Structures of the Newly Designed Diketo Hexenoic derivatives **5a**-**m**, **6a**-**m**, **7a**-**d**, and **8a**-**d** and Diketo Butanoic derivatives **9a**,**b**, **10a**,**b**, **11a**,**b** and **12a**,**b**.

Therefore, we designed and synthesised novel diketo hexenoic derivatives **5a**-**m**, **6a**-**m**, **7a**-**d**, and **8a**-**d** and diketo butanoic derivatives **9a**,**b**, **10a**,**b**, **11a**,**b** and **12a**,**b** with improved inhibition potency against TdT (Figure 4).

## Materials and Methods

### Chemistry

#### General instrumentation

Melting points were determined on a Bobby Stuart Scientific SMP1 melting point apparatus and are uncorrected. Compound purity was always >95% as determined by combustion analysis. Analytical results agreed to within ± 0.40% of the theoretical values. IR spectra were recorded on a PerkinElmer Spectrum-One spectrophotometer.^1^H NMR spectra were recorded at 400 MHz on a Bruker AC 400 Ultrashield 10 spectrophotometer (400 MHz). Dimethyl sulfoxide-*d*_6_ 99.9% (CAS 2206–27-1) and Chloroform-*d_3_* 98.8% (CAS 865–49-6) of isotopic purity (Aldrich) were used. Column chromatographies were performed on silica gel (Merck; 70 − 230 mesh). All compounds were routinely checked on TLC by using aluminium-baked silica gel plates (Fluka DC-Alufolien Kieselgel 60 F_254_). Developed plates were visualised by UV light. Solvents were reagent grade and, when necessary, were purified and dried by standard methods. Concentration of solutions after reactions and extractions involved the use of rotary evaporator (Büchi) operating at a reduced pressure (ca. 20 Torr). Organic solutions were dried over anhydrous sodium sulphate (Merck). All solvents were freshly distilled under nitrogen and stored over molecular sieves for at least 3 h prior to use.

#### General experimental procedures

**General procedure A (GP-A) to obtained Pyrroles 13c-e,k**. To a solution of dry *N,N*-dimethylformamide (DMF) (81.9 mmol) in dry 1,2-dichloroethane (18 ml) cooled into ice bath, was added dropwise a solution of oxalyl chloride (81.9 mmol) in dry 1,2-dichloroethane (12 ml) within 20 min. After the addition, the suspension that formed was stirred at room temperature for 15 min. The mixture was placed back into ice bath, and then a solution of pyrrole (74.5 mmol) in dry 1,2-dichloroethane (15 ml) was added dropwise within 20 min. The reaction mixture was stirred at room temperature for 15 min, then AlCl_3_ (163.9 mmol) and the appropriate benzoyl chloride (74.5 mmol) were added. The reaction mixture was stirred at room temperature for 3 h 40 min, treated with crushed ice and water (745 ml) and then a solution of 50% NaOH (60 ml) was added before a further stirring of 10 min. The aqueous phase was acidified with 1 N HCl until pH 4 was reached, the obtained precipitate was collected by filtration, washed with water, and light petroleum ether to obtain pure derivatives **13c-e,k**. For each compound, proper benzoyl chloride, chromatographic system, yield (%), melting point (°C), IR,^1^H NMR, and elemental analyses are reported in the Supplementary Materials.

**General procedure B (GP-B) to obtain *N*-Alkylated Pyrrolyl Derivatives 14c-f,h-j.** A mixture of the appropriate pyrrole (1.1 mmol), alkylating agent (3.3 mmol), and anhydrous K_2_CO_3_ (1.5 mmol) in dry DMF (10 ml) was stirred at 100 °C for the appropriate time. Then the mixture was cooled, treated with water (40 ml), and extracted with ethyl acetate. Alternatively, for compound **14j**, after treatment with water, the solid that formed was filtered, washed with diethyl ether (2 × 10 ml), and with light petroleum ether (2 × 20 ml) to obtain pure compound. The organic layer was washed with brine, dried over anhydrous sodium sulphate, and concentrated under vacuum. The crude product was purified by chromatography on silica gel to afford the pure product. For each compound, alkylating agent, reaction time, chromatographic system, yield (%), melting point (°C), IR, ^1^H NMR, and elemental analyses are reported in the Supplementary Materials.

**General Procedure C (GP-C) to obtain α,β-Unsaturated Derivatives 15c-k,m.** The proper pyrrole carboxaldehyde (0.075 mol) was dissolved in 250 ml of acetone. To this mixture, 4 N NaOH (110 ml) was added, and the mixture was stirred at room temperature for 24 h. Water (300 ml) and ethyl acetate (250 ml) were added, the organic layer was separated, washed with water (2 × 100 ml), dried over sodium sulphate, filtered, and evaporated under reduced pressure. The crude product was purified by column chromatography on silica gel to obtain pure products. For each compound, chromatographic system, yield (%), melting point (°C), IR, ^1^H NMR, and elemental analyses are reported in the Supplementary Materials.

**General Procedure D (GP-D) to obtain Diketo Esters 5c-k,m and 7c,d.** For compounds **5c-k,m**, freshly prepared sodium ethoxide, obtained by the dissolution of Na (63 mmol) in 56 ml of absolute ethanol, was added to a well-stirred solution of the proper α,β-unsaturated ketone derivative (31 mmol) and diethyl oxalate (62 mmol) in dry tetrahydrofuran (THF) (31 ml) under argon atmosphere.

For compounds **7c,d**, freshly prepared sodium ethoxide, obtained as above, was added to a well-stirred solution of the proper acetyl derivative (31 mmol) in dry THF (31 ml) under argon atmosphere; afterwards, diethyl oxalate (62 mmol) was added.

The mixture was stirred at room temperature for 1 h 30 min under argon atmosphere and then was poured into *n*-hexane (704 ml). The resulting precipitate was vigorously stirred for 30 min in 1 N HCl (704 ml). The solid that formed was filtered, washed with water and light petroleum ether, and dried under IR lamp to afford the pure diketo esters. For each compound, yield (%), melting point (°C), IR, ^1^H NMR, and elemental analysis are reported in the Supplementary Materials.

**General Procedure E (GP-E) to obtain Diketo Acids 6c-k,m and 8c,d.** A mixture of 1 N NaOH (9.5 ml) and the appropriated ester (1.9 mmol) in 1:1 THF-methanol (9.3 ml) was stirred at room temperature for 30 min and then poured into crushed ice. The mixture was treated with 1 N HCl until pH 3 was reached and extracted with ethyl acetate (3 × 100 ml). The collected organic extract was washed with brine (3 × 100 ml), and dried, and the solvent was evaporated under reduced pressure to give the pure diketo acids. For each compound, yield (%), melting point (°C), IR, ^1^H NMR, and elemental analyses are reported in the Supplementary Materials.

^1^H NMR and FTIR Spectra for the newly synthesised compounds **5c-k,m**, **6c-k,m 7c-d**, **8c-d** are reported in Supplementary Materials as Figures S3–S50.

#### Specific experimental Procedures and characterization

Specific experimental procedures and characterisation of compounds **5a**-**m**, **6a**-**m**, **7a**-**d**, **8a**-**d, 9a**,**b**, **10a**,**b**, **11a**,**b** and **12a**,**b** are reported in Supplementary Material.

### Biological assays

#### Enzymes and proteins

Recombinant full-length human DNA polymerase λ and β were generated and purified as described^29^. After purification, the protein was >90% homogeneous, as judged by sodium dodecyl sulphate (SDS)−polyacrylamide gel electrophoresis (PAGE) and Coomassie staining. TdT was from Trevigen (Gaithersburg, MD).

#### Inhibition assays

*Polymerase Assay*: Activity of DNA polymerase λ and β were assessed using poly(dA)/oligo(dT)10:1 substrate in a final volume of 25 μL containing 50 mM Tris-HCl (pH 7.0), 0.25 mg/mL BSA, 1 mM, DTT, 0.2 μM poly(dA)/oligo(dT)10:1 (3′-OH ends), 50 nM DNA polymerase λ or β, 5 μM [3H]-2′-deoxythymidine 5′-triphosphate (dTTP) (5 C_i_/mmol), and 0.5 mM MnCl_2_ or 5 mM MgCl_2_ used as cofactors. The polymerase activity was monitored in the presence of 10% DMSO or in the presence of increasing amounts of inhibitors dissolved in DMSO. All reactions were incubated for 15 min at 37 °C and the DNA precipitated with 10% trichloroacetic acid. Insoluble radioactive material was determined by scintillation counting using Trilux beta-counter (PerkinElmer) as described^30^.

*Terminal Transferase Assay*. DNA polymerase λ and TdT terminal transferase activities were assayed in a final volume of 25 μL containing 50 mM Tris-HCl (pH 7.0), 0.25 mg/mL BSA, 1 mM DTT, 0.2 μM ss 30mer (AAC)_10_ DNA oligonucleotide, 25 μM [3H]dNTPs (10 Ci/mmol) and 0.5 mM MnCl_2_ or 5 mM MgCl_2_ used as cofactors. Terminal transferase activity was monitored in the presence of 10% DMSO or in the presence of increasing amounts of inhibitors dissolved in DMSO. All reactions were incubated for 10 min at 37 °C and the DNA precipitated with 10% trichloroacetic acid. Insoluble radioactive material was determined by scintillation counting using Trilux beta-counter (PerkinElmer) as described^26^.

Dose − response curves were generated by computer fitting of the data to the equation E_(%)_ = E*_max_*/(1 + I/ID_50_) where E_(%)_ is the fraction of enzyme’s activity measured in the presence of the inhibitor, E*_max_* is the activity in the absence of the inhibitor, I is the inhibitor concentration, and ID_50_ is the inhibitor concentration at which E_(%)_ = 0.5 E*_max_*. ID_50_ values were generated using GraphPad Prism 6.

### Docking studies

All molecular modelling studies were performed on a MacPro dual 2.66 GHz Xeon running Ubuntu 20 LTS. The human TdT primary aminoacidic sequence was derived from Uniprot (UniProtKB: P04053). The homology model was carried out by Prime module^31^ of Maestro^32^. The enzyme *Mus Musculus* ortholog was available at the protein data bank server (PDB code: 4IQW)^28^. The coordinates of nucleic acid and sodium atom was from PDB 4IWQ structure. The coordinates of Mg^2^**^+^** and Mn^2+^ atoms were from PDB 4I2B and 4I2C structure, respectively^33^. Hydrogen atoms were added to the protein, using Maestro protein preparation wizard^34^. The obtained structure was minimised to avoid steric clash by minimisation module of Maestro. A constraint was applied to all heavy atoms. This structure, modelled as described above, was then validated by creating the related Ramachandran plot (Figure S1 Supplemental material). Ligand structures were built with Maestro and minimised using the MMFF94x force field until a rmsd gradient of 0.05 kcal/(mol·Å) was reached. The docking simulations were performed using Plants^35^ using all default settings and a radius of 12 Å. The images in the manuscript were created with PyMOL^36^.

## Results and discussion

### Chemistry

**Scheme 1.**
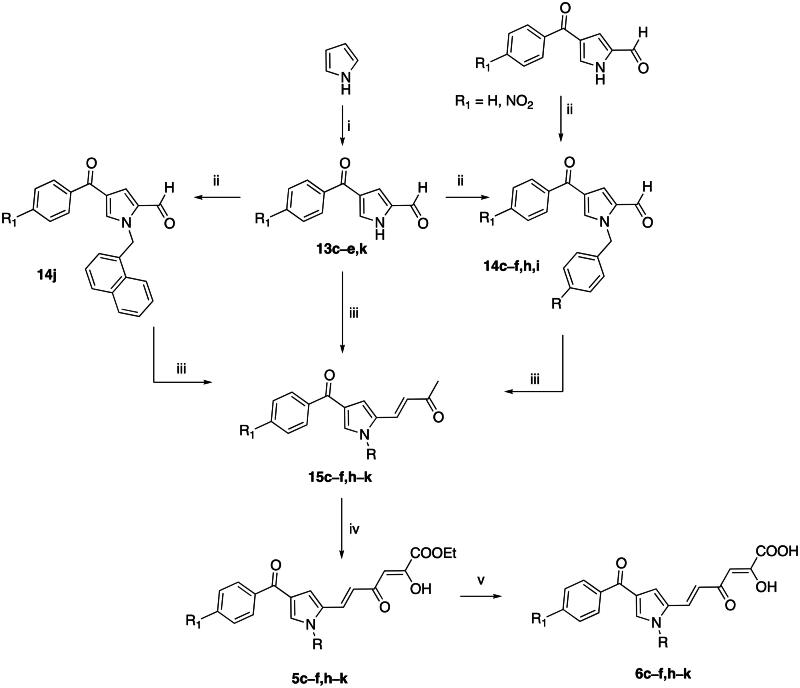
Synthetic Route to Compounds **5c**-**f,h-k** and **6c-f,h-k.** Reagents and conditions: (i) (1) dry DMF, oxalyl chloride, dry 1,2-dichloroethane, 0 °C to room temp, 20 min; (2) appropriate benzoyl chloride, AlCl_3_, room temp, 4 h 35 min, 50–74% yield; (ii) appropriate alkylating agent, K_2_CO_3_, dry DMF, 100 °C, 4–8 h, 60 − 82% yield; (iii) acetone, NaOH 4 N, room temp, 24 h, 65–74% yield; (iv) diethyl oxalate, NaOEt, dry THF, Ar, room temp, 2 h, 68–75% yield; (v) NaOH 1 N, THF/methanol 1:1, room temp, 30 min, 85–98% yield.

Compounds **5a**,**b**,**l** and the corresponding acids **6a,b,l** were synthesised as reported in literature^37–41^. Pyrrole derivatives **5c-f,h-k** and **6c-f,h-k** were instead synthesised as reported in Scheme 1. 1*H*-Pyrrole underwent a two-steps one-pot Vilsmeyer-Haack formylation and Friedel-Crafts acylation reactions: the first step was carried out with oxalyl chloride in DMF, followed by the addition of the appropriate aroyl chloride (commercially available) and aluminium trichloride as Lewis acid, obtaining the corresponding the 2,4-disubstituted pyrroles **13c-e,k**.

Carbaldehydes **13c-e,k**, 4-benzoyl-1*H*-pyrrole-2-carbaldehyde^28^, and 4–(4-nitrobenzoyl)-1*H*-pyrrole-2-carbaldehyde^42^ were alkylated with the proper benzyl bromide (or 2-(bromomethyl)naphthalene for derivative **14j**) in alkaline medium to give the *N*-substituted pyrroles **14c-f,h-j**. The NH aldehyde **13k** and *N*-substituted aldehyde intermediates **14c**-**f,h-j** underwent a condensation reaction with acetone in the presence of NaOH to give the corresponding α,β-unsaturated ketones **15c**-**f,h-k**. Then, the enones **15c**-**f,h-k** were reacted with diethyl oxalate according to a Claisen-Schmidt condensation reaction, in presence of sodium ethoxide as base, to afford the corresponding diketo esters **5c**-**f,h-k**. Final alkaline hydrolysis of these esters led to diketo acids **6c**-**f,h-k**.

**Scheme 2.**
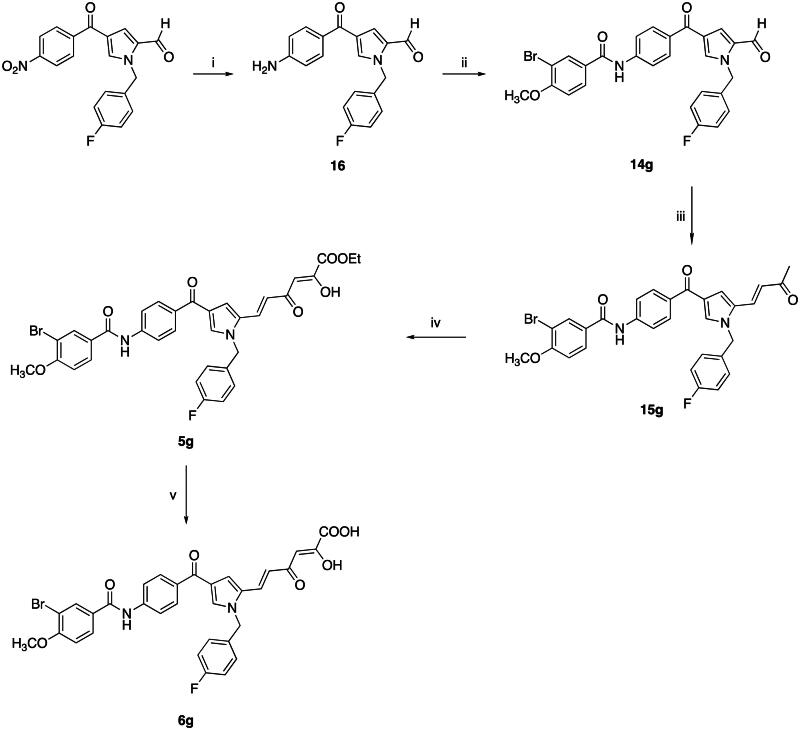
Synthetic Route to Compounds **5g** and **6g.** Reagents and conditions: (i) ammonium formate, NHEt_2_, Pd/C 10%, AcOEt, Ar, reflux, 1 h, 100% yield; (ii) 3-bromo-4-methoxybenzoyl chloride, Et_3_N, dry CH_2_Cl_2_, Ar, 0 °C to room temp, 15 h, 35% yield; (iii) acetone, NaOH 4 N, room temp, 24 h, 74% yield; (iv) diethyl oxalate, NaOEt, dry THF, Ar, room temp, 2 h, 68% yield; (v) NaOH 1 N, THF/methanol 1:1, room temp, 30 min, 85% yield.

The synthesis of compounds **5g** and **6g** resembled the one described above and is reported in Scheme 2. More in detail, compound 4–(4-nitrobenzoyl)-1*H*-pyrrole-2-carbaldehyde^42^ underwent a nitro group reduction according to Leuckart reaction with ammonium formate and diethylamine using palladium on carbon^43^. The corresponding amine **16** was used in the following amidation reaction with commercial 3-bromo-4-methoxybenzoyl chloride using triethylamine as a base to afford compound **14g**. The subsequent crossed aldol condensation with acetone, Claisen-Schmidt and alkaline hydrolysis gave compounds **15g**, **5g** and **6g**, respectively, in a similar fashion to the previous scheme.

**Scheme 3.**
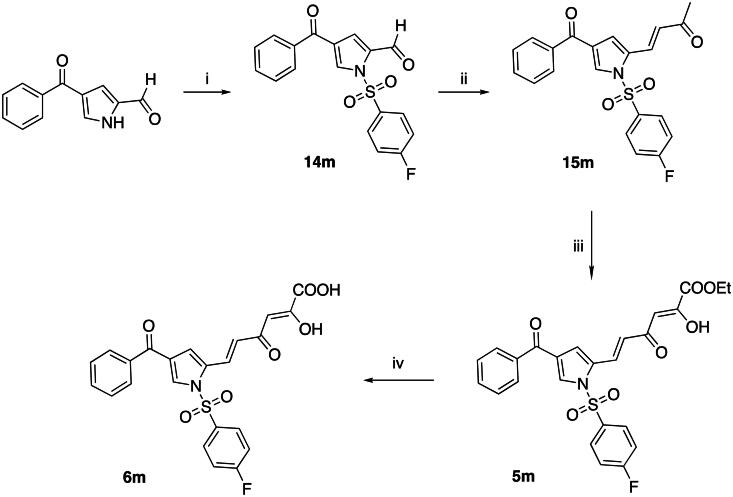
Synthetic Route to Compounds **5m** and **6m.** Reagents and conditions: (i) 4-fluorobenzene-1-sulphonyl chloride, NaH (60% dispersion in mineral oil), dry THF, 0 °C to room temp, 12 h, 88% yield; (ii) acetone, NaOH 4 N, room temp, 24 h, 68% yield; (iii) diethyl oxalate, NaOEt, dry THF, Ar, room temp, 2 h, 73% yield; (iv) NaOH 1 N, THF/methanol 1:1, room temp, 30 min, 90% yield.

The sulphonyl derivatives **5m** and **6m** have been synthesised as reported in Scheme 3. By reaction of 4-benzoyl-1*H*-pyrrole-2-carbaldehyde^28^ with 4-fluorobenzene-1-sulphonyl chloride in presence of NaH, the *N*-sulphonyl derivative **14m** was obtained. The subsequent crossed aldol condensation with acetone, Claisen-Schmidt condensation and alkaline hydrolysis gave compounds **15m**, **5m** and **6m**, respectively, as described in the previous schemes.

**Scheme 4.**
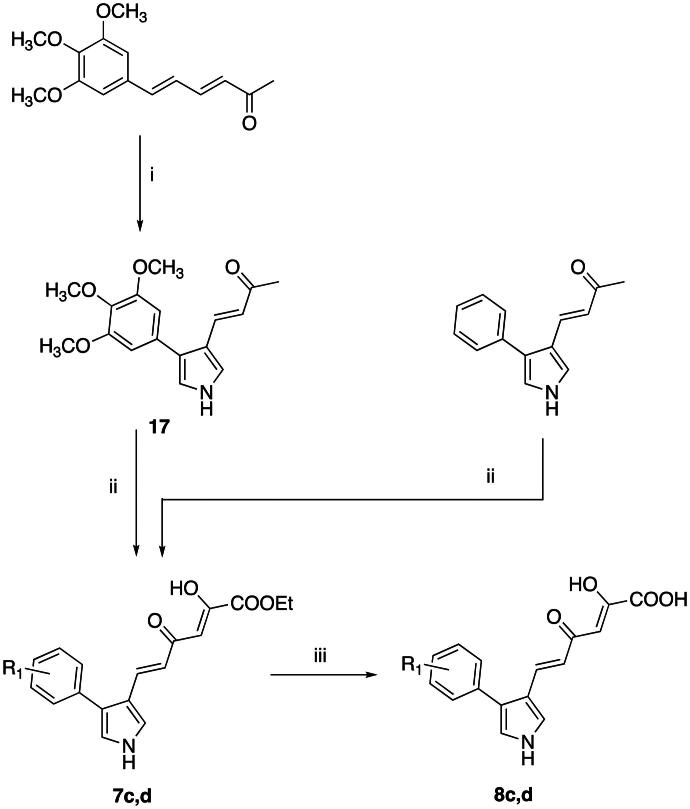
Synthetic Route to Compounds **7c**,**d** and **8c**,**d**. Reagents and conditions: (i) TosMIC, NaH (60% dispersion in mineral oil), dry diethyl ether/DMSO 2:1, Ar, room temp, 30 min, 62% yield; (ii) diethyl oxalate, NaOEt, dry THF, Ar, room temp, 2 h, 70–75% yield; (iii) NaOH 1 N, THF/methanol 1:1, room temp, 30 min, 90% yield.

The 3-diketo derivatives **7a**,**b** and **8a**,**b** were synthesised as reported in literature^38^^,^^40^ while esters **7c**,**d** and their corresponding acids **8c**,**d** were synthesised according to Scheme 4. The intermediate (*3E*,*5E*)-6–(3,4,5-trimethoxyphenyl)hexa-3,5-dien-2-one^44^ underwent a Van Leusen ring closure by reacting with TosMIC, giving the pyrrole derivative **17**. Then, the enones **17** and (*E*)-4–(4-phenyl-1*H*-pyrrol-3-yl)but-3-en-2-one^38^ were reacted with diethyl oxalate to afford esters **7c**,**d**. Final hydrolysis of these esters led to diketo acids **8c**,**d**.

Lastly, the diketo butanoic derivatives **9a**,**b**, **10a**,**b**, **11a**,**b** and **12a**,**b**, were obtained as previously described^38^^,^^40^. The detailed synthetic procedures and the analytical and spectroscopic data of the synthesised compounds are reported in the Materials and Methods section.

### In vitro enzymatic assay

#### Diketo hexenoic and diketo butanoic derivatives are highly active towards TdT

The newly synthesised compounds **5a**-**m**, **6a**-**m**, **7a**-**d**, **8a**-**d**, **9a**,**b**, **10a**,**b**, **11a**,**b** and **12a**,**b** were tested against the family X enzyme TdT. The results are indicated in Table 1.

All DNA polymerases can perform DNA synthesis using divalent metal cations Mg^2+^ or Mn^2+^ as cofactors^45^, with TdT also able to utilise other metal ions such as Ca^2+^ and Co^2 + ^29^,^^46^. Due to different steric hindrance, coordination of DNA-dependent polymerases active site of by Mg^2+^ or Mn^2+^ differently affected reaction efficiency and fidelity^45^. In mammalian cells, the concentration of Mg^2+^ ranges from 1 to 20 mM, while Mn^2+^ is present at lower concentrations, spamming between 0.01 and 0.2 mM^47^. However, complex formation with Mn^2+^ or Mg^2+^ ions depends on the affinity of the specific polymerase for each metal ion. As instance, pol λ exhibits higher affinity towards Mn^2+^, suggesting that at least family X members exists in cells associated with different metal cofactors^48^. For this reason, evaluating the influence of the metal cofactor on the potency of inhibition and selectivity towards specific polymerase of newly synthesised diketo hexenoic derivatives **5a-m, 6a-m, 7a-d, 8a-d** and diketo butanoic derivatives **9a,b, 10a,b, 11a,b** and **12a,b** is essential to correctly predict their activity in the cellular environment. For this reason, all tested compounds were evaluated in inhibition assays performed using either Mg^2+^ or Mn^2+^ as cofactors. When evaluated in the presence of Mg^2+^, 8 compounds were active in the submicromolar range, 7 compounds were active in the low micromolar range, and 7 compounds showed lower activity (10 μM < ID_50_ < 50 μM). Only 2 compounds were completely inactive (ID_50_ > 50 μM). When reactions were conducted in presence of Mn^2+^, 6 compounds were active in the submicromolar range, 12 compounds proved to be active in the low micromolar range, and 10 compounds resulted inactive (ID_50_ > 50 μM). In general, it is possible to notice that the acid derivatives proved to be more active than the ester counterparts both in presence of Mg^2+^and Mn^2+^ions.

When compounds were tested in the presence of Mg^2+^ion, among the *N*-*p*-fluorobenzyl substituted derivatives (**5a-g** and **6a-g**), it is possible to notice that the presence of a benzoyl group in position 4 of the pyrrole ring led to an improvement of activity. Indeed, compounds **6a** and **6b**, characterised respectively by an H or a phenyl group in position 4, reported an activity higher than 50 μM, while the 4-benzoyl substituted derivatives showed ID_50_ values within 0.41 − 12.76 μM. More in detail, a group characterised by both steric hindrance and a balance between hydrophilicity and lipophilicity benefits the activity, both in the case of esters and the acids. In particular, by introducing the 4–(3-bromo-4-methoxybenzamido)-benzoyl substituent, the best inhibitory values were observed (**5g**: ID_50_ = 0.91 μM; **6g**: ID_50_ = 0.41 μM). On the other hand, the introduction of a naphthalen-2-ylmethoxy or of a nitro group showed less promising results. Differently, by keeping fixed the 4-benzoyl substituent (compounds **5h-j** and **6h-j**), the presence of a steric hindered and electron-donating group led to an improvement of inhibitory activity. Indeed, the trend of activity varies in the following order: napht-1-ylmethyl (**5j**) > methoxy (**5i**) > chlorine (**5h**) for the ester derivatives (**5j**: ID_50_ = 3.68 μM; **5i**: ID_50_ = 10.54 μM; **5h**: ID_50_ = 17.51 μM), and napht-1-ylmethyl (**6j**) ≈ methoxy (**6i**) > chlorine (**6h**) for the acid derivatives (**6j**: ID_50_ = 2.11 μM; **6i**: ID_50_ = 2.70 μM; **6h**: ID_50_ = 5.10 μM). For NH derivatives, the presence of benzoyl substituent or a phenyl one in position 4 of the pyrrole ring seems to not affect the activity both for acid and ester derivatives (benzoyl derivatives **5k,l** and **6k,l** and phenyl derivatives **7c,d** and **8c,d**). In detail, for ester derivatives, the presence of an unsubstituted benzoyl group led to ID_50_ value of 37.82 μM (**5l**), while the corresponding phenyl derivative reported ID_50_ value of 10.5 μM (**8c**). Similar trend was observed for the 3,4,5-trimethoxybenzoyl and 3,4,5-trimethoxyphenyl derivatives (**5k**: ID_50_ = 10.61 μM; **7d**: ID_50_ = 21.29 μM). For the acid counterparts, a comparable behaviour was reported, with the exception of the 3,4,5-trimethoxyaryl derivatives (**6k**: ID_50_ = 0.86 μM; **8d**: ID_50_ = 0.24 μM; **6l**: ID_50_ = 0.22 μM; **8c**: ID_50_ = 0.11 μM). Moreover, the presence of an unsubstituted aryl ring in position 4 seems to be more promising for the inhibition.

In the presence of Mn^2+^ion, considering the *N*-*p*-fluorobenzyl substituted derivatives (**5a-g** and **6a-g**), ester compounds showed ID_50_ values higher than 50 μM while acid derivatives showed activities within 1.63 − 7.71 μM. Among them, **6a**, which is characterised by the presence of a H in position 4 of the pyrrole ring was the best compound, showing an ID_50_ of 1.63 μM, 4 times higher than its 4-phenyl substituent counterpart **6b**. Notably, **6a** proved to be about 6 times more active than the hit compound **2**. It is worthy of note that by shifting the diketo hexenoic chain from position 2 to position 3 of the pyrrole core, an opposite trend was observed. In particular, the presence of a phenyl group in position 4 led to better results (4-Ph substituted derivatives **7b** and **8b** *vs* 4-H substituted compounds **7a** and **8a**, respectively; **7b**: ID_50_ = 2.72 μM; **8b**: ID_50_ = 0.66 μM; **7a**: ID_50_ > 50 μM; **8a**: ID_50_ = 6.31 μM). Considering the diketo butanoic derivatives (**10a,b** and **12a,b**) the presence of phenyl group in position 4 led to less interesting results in respect to the 4-H substituted counterparts, both in the case of 2-diketo hexenoic and 3-diketo hexenoic derivatives. Noteworthy, the replacement of the CH_2_ of the benzyl ring with a SO_2_ group clearly improved the inhibitory activity of both esters and acids. This can be observed comparing SO_2_-substituted derivatives **5m** and **6m** with CH_2_-substituted counterparts **2-COOEt** (data not shown^28^), and **2** (**5m**: ID_50_ = 8.75 μM; **6m**: ID_50_ = 0.33 μM; **2-COOEt**: ID_50_ > 40 μM; **2**: ID_50_ = 9.50 μM). Importantly, compounds **5m** and **6m** are among the most active compounds against TdT also when Mg^2+^was used as co-factor (**5m**: ID_50_ = 3.87 μM; **6m**: ID_50_ = 0.34 μM). On the other hand, by keeping fixed the 4-benzoyl substituted derivatives, the presence of a 4-fluorobenzyl group seems to be optimal for the activity for both esters and acids. Considering NH derivatives, the presence of benzoyl substituent (**5k,l** and **6k,l**) or a phenyl one (**7c,d** and **8c,d**) differently affected the inhibitory potency. More in detail, compound **5l**, characterised by an unsubstituted benzoyl group, showed a ID_50_ value higher than 50 μM, similarly to its corresponding phenyl counterpart **7c** and 3,4,5-trimethoxyphenyl compound **7d**. On the contrary, acid compounds **6k** and **6l** seems to be not affected by the presence of a benzoyl or 3,4,5-trimethoxybenzoyl group, showing comparable activities, (**6l**: ID_50_ = 0.30 μM; **6k**: ID_50_ = 0.38 μM). Compared to hit compounds **3** and **4**, compounds **6k** and **6l** also showed potencies, in the submicromolar range, with compound **6 l** being the most active NH derivative. Interestingly, ester derivative **5k** showed 3 times lower potency than its acid counterpart **6k**. Acid derivatives **8c** and **8d** were among the more active compounds. They inhibit TdT in the submicromolar range, being not affected by the presence of a phenyl or a 3,4,5-trimethoxyphenyl group (**8c**: ID_50_ = 0.19 μM; **8d**: ID_50_ = 0.23 μM).

In general, considering the results in presence of both Mg^2+^and Mn^2+^ions, diketo acids derivatives **6k-m** and **8c,d**, which showed the best TdT inhibitory potencies, were proved to be the best inhibitors found.

**Figure 5.**
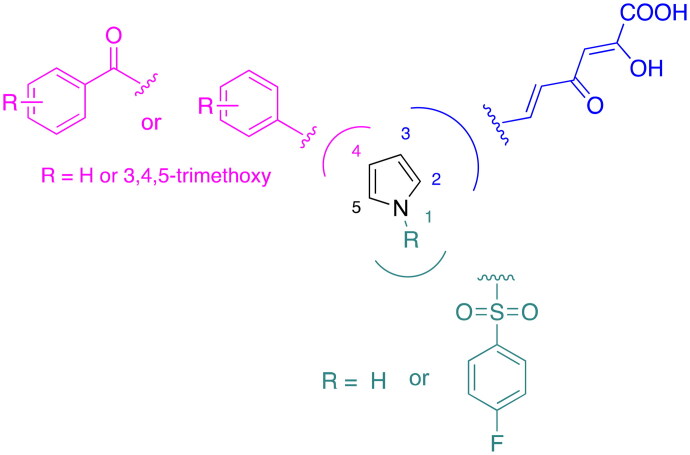
Chemical features of pyrrolyl DKAs modulating their anti-TdT properties.

Altogether, we were then able to describe the structural features that a compound should hold to achieve TdT inhibition in the presence of Mg^2+^ or Mn^2+^ ions (Figure 5).

#### Diketo hexenoic and diketo butanoic derivatives are selective towards TdT

To evaluate their selectivity, the newly synthesised compounds were also tested against the other X family members pol λ and pol β. Diketo derivatives have a clear preference for TdT inhibition, showing low to negligible activity towards both pol λ and pol β (Table S1). When tested at a fix concentration of 100 µM, only four diketo hexenoic derivatives (**5h,i** and **6h,i**) showed less than 10% residual polymerase activities. For these compounds, ID_50_ was calculated (Table 2). Pol λ, besides synthesising DNA in a template-dependent manner, is also able to act as a terminal transferase, elongating a ssDNA primer. This activity is greatly favoured when the enzyme is in association with Mn^2+^.^49^ Nonetheless, none of these compounds (**5h,i** and **6h,i**) were active against TdT-like activity of pol λ, while showing only modest potency towards the canonical template-dependent activity of both pol λ and pol β. Interestingly, none of these molecules are potent inhibitors of TdT, with **6i** (ID_50_ = 2.7 µM) being the most active.

More in detail, the acid derivatives, **6h,i**, showed weak potencies against the template-dependent polymerase activity of pol λ, comparable or lower than that against TdT (**6h**: ID_50_ pol λ (Pol) = 4.06 μM, ID_50_ TdT = 5.10 μM; **6i**: ID_50_ pol λ (Pol) = 4.06 μM, ID_50_ TdT = 2.70 μM). Similarly, the ester counterparts (**5h,i**) are moderate inhibitors of the template-dependent polymerase activity of pol λ, showing less selectivity than their acid analogues against TdT (**5h**: ID_50_ pol λ (Pol) = 4.08 μM, ID_50_ TdT = 17.51 μM; **5i**: ID_50_ pol λ (Pol) = 4.07 μM, ID_50_ TdT = 10.54 μM). Interestingly, the newly synthesised compounds showed a different pattern of inhibition with respect to the hit compounds, which inhibited both TdT and the TdT-like activity of DNA pol λ.

The same compounds were active also against pol β, but with a lower extent than towards pol λ (Table 2). More in detail, diketo hexenoic compounds **5h,i** showed ID_50_ values *vs* pol β lower than that against TdT (**5h**: ID_50_ pol β = 12.36 μM, ID_50_ TdT = 17.51 μM; **5i**: ID_50_ β = 7.94 μM, ID_50_ TdT = 10.54 μM). On the other hand, the acid counterparts, **6h,i**, showed higher potencies against TdT in respect to the polymerase activity of pol λ (**6h**: ID_50_ pol β = 19.95 μM, ID_50_ TdT = 5.10 μM; **6i**: ID_50_ pol β = 14.67 μM, ID_50_ TdT = 2.70 μM).

### Docking studies

The newly synthesised derivatives were studied by docking experiments to gain more insight into the compounds binding mode and to identify the pharmacophoric moieties. The human TdT structure was not available in Protein Data Bank, thus a homology model approach was carried out using as reference the *Mus Musculus* ortholog. The homology between the two structures is close to 80% for the full length and rich value close to 100% about the catalytic site. Derivatives **3** and **4** were previously co-crystallized^28^, and the TdT structure complexed with **4** was used as reference to better understand the binding modes of our newly synthesised compounds. The model was prepared as reported in Materials and Methods section (§2.3), taking into account the presence of catalytic ions Mg^2+^ or Mn^2+^. Worthily, the proposed binding poses with either ions are perfectly superimposable, thus underlying a common nucleotide competitive mechanism of action (Figure S2 Supplemental material).

**Figure 6.**
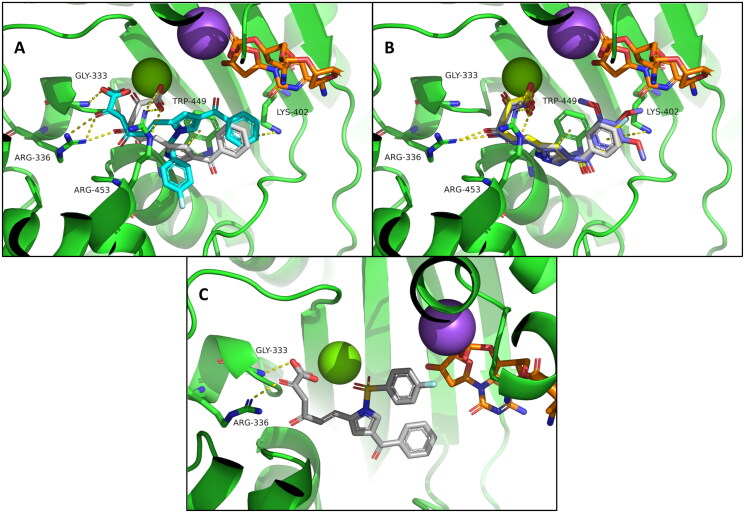
Proposed binding mode for benzoyl derivatives (A) **2** (cyan) and **6l** (white); (B) **4** (yellow), **6k** (light blue) and **6l** (white); (C) **6m** (light grey). The enzyme is depicted as green cartoon; nucleic acid is reported as orange sticks; sodium and Mg^2+^are reported as purple and green spheres respectively.

**Figure 7.**
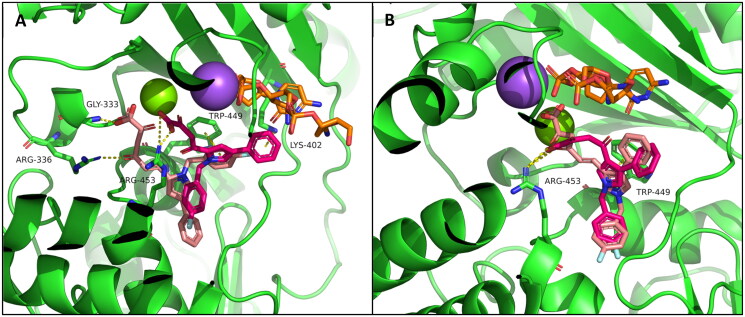
Proposed binding mode for phenyl derivatives (A) **6b** (pink) and **10b** (magenta); (B) **8b** (pink) and **12b** (magenta). The enzyme is depicted as green cartoon; nucleic acid is reported as orange sticks; sodium and Mg^2^**^+^** are reported as purple and green spheres, respectively.

Then, all reported derivatives were submitted to docking studies. The studied compounds can be divided into two different patterns of substitution on position 4 of the pyrrole ring: the benzoyl derivatives, which can be NH or *N-*substituted with a DKA chain in position 2, and the phenyl derivatives, characterised by a *p*-fluorobenzyl group in position 1 and a DKA chain in position 2 or 3. Despite the substitutions, the compounds share a consistent binding mode, and the interactions of the pyrrole and the DKA moieties are retained. About the pyrrole ring, it can interact *via* π-π stacking with Trp449 (Figures 6(A,B), 7( A–B)). For the DKA chain is observed that the ketone moiety can form a H-bond with Arg336 (Figures 6(A–C), 7( A)) whereas the carboxylic function has polar contacts with the backbone of Gly333 (Figure 6(A,C)) and with Arg453 (Figures 6(A–C), 7( A–B)). Also, the generated binding modes show how the DKA chain is favourably oriented to allow the chelation of the catalytic ion. This is not surprising, as DKA group is a well-known chelating moiety. Moreover, chelation by the carboxylate group should explain the higher inhibitory activity displayed by acid compounds compared to the corresponding esters, despite sharing the same predicted binding position in the TdT binding site (data not shown).

Among the benzoyl derivatives, the introduction of the benzyl group to the pyrrole nitrogen atom does not overly affect the binding mode and the already described interactions established by DKA chain were retained (Figure 6(A)). However, the introduction of this bulky group, which does not establish additional interactions, decreases the degrees of freedom of the molecule, making more difficult the π-π stacking between the pyrrole ring and Trp449. Indeed, the NH derivative shows better activities than the benzyl derivative (**2**: ID_50_ Mg^2+^ = nd; ID_50_ Mn^2+^ = 9.50 μM; **6 l**: ID_50_ Mg^2+^ = 0.22 μM; ID_50_ Mn^2+^ = 0.30 μM).

For NH derivatives, the presence of benzoyl substituent seems to not excessively affect the activity (**4**: ID_50_ Mg^2+^ = nd; ID_50_ Mn^2+^ = 0.58 μM; **6k**: ID_50_ Mg^2+^ = 0.86; ID_50_ Mn^2+^ = 0.36 μM; **6l**: ID_50_ Mg^2+^ = 0.22 μM; ID_50_ Mn^2+^ = 0.30 μM). All these derivatives show a superimposable binding pose, in which the benzoyl group establishes a cation-π interaction with Lys402. Cation-π interactions are usually influenced by their repulsion, electrostatic, induction, dispersion, and many other contributions, such as the steric one^50^. It is likely that the electron donor effect of the methoxy groups in compound **6k** does not lead to a significant increase in activity because of the excessive steric bulk. The presence of fluorine atom as electron-withdrawing substituent in compound **4**, on the other hand, slightly decreases the activity compared with the unsubstituted benzoyl derivative **6l**.

Worthily, **6m** shows an interesting binding mode in which the sulphone moiety angling itself to allow the ion chelation (Figure 6C). Interestingly, *in silico* predictions are in agreement with the biological data (**6m**: ID_50_ Mg^2+^ = 0.34 μM; ID_50_ Mn^2+^ = 0.34 μM).

Regarding the phenyl derivatives, these compounds are characterised by a *p*-fluorobenzyl group in position 1 of the pyrrole ring and a DKA chain in position 2 or 3.

In Figure 7(A) are reported the docking outputs for compounds **6b** and **10b**, respectively characterised by a diketo hexenoic and butanoic chain in position 2. The shorter chain of **10b** allows the molecule to better chelate the ion and better fit in the binding pocket, leading to a π-π stacking between the pyrrole ring and Trp449 and a cation-π interaction between the phenyl ring and Lys402. **6b** not only shows a less effective orientation for chelation, but also a lower number of interactions with the protein.

The shifting of the DKA chain from position 2 to 3 did not affect the binding mode respect to **10b** and neither the type of interactions (Figure 7(B)), even if in this case the diketo hexenoic chain of **8b** can better reach the ion, establishing a more efficient chelation (**8b**: ID_50_ Mg^2+^ = nd; ID_50_ Mn^2+^ = 0.66 μM; **12b**: ID_50_ Mg^2+^ = nd; ID_50_ Mn^2+^ = 10.10 μM).

## Conclusions

Here we report the structure-activity relationships studies of new pyrrolyl-DKA derivatives as structural analogues of our previously reported TdT inhibitors. Thanks to the results shown here, we were able to rationalise the structural requirements to inhibit the enzymatic target. Interestingly, acid derivatives proved to be more active than the ester counterparts. Moreover, the introduction of a diketo hexenoic chain in position 2 or 3 of the pyrrole core led to more promising results. In general, it can also be highlighted that NH or *N-*sulphonyl substituted compounds (**6k-m**, **8c,d**) reported the best inhibitory potencies, data also corroborated by docking studies that rationalised the basis of such improvement in activity. Interestingly, most of the synthesised compounds showed low micromolar/submicromolar inhibitory activities against TdT, proving also to be selective in respect to pol λ and pol β. Importantly, the best acting compound **8c** reported a marked improvement (higher than 3 times) in the inhibitory activity in respect to the hit compound **4**. In conclusion, the results shown here give important insights for a future optimisation within this class of compounds.

## Supplementary Material

Supplemental_TdT_Inhibitors_Madia_et_al_ID_251780404_R1_CLEAN_COPY.docx

Table S1.docx

## Data Availability

The data presented in this study are available on request from the corresponding author.
