# Supplementary material for: Exploring structure–activity relationships of pyrrolyl diketo acid derivatives as non-nucleoside inhibitors of terminal deoxynucleotidyl transferase enzyme
Source: J Enzyme Inhib Med Chem. 2025 Jun 9;40(1):2496782. doi: 10.1080/14756366.2025.2496782 (PMC12150604; doi:10.1080/14756366.2025.2496782)
Supplement: Supplemental_TdT_Inhibitors_Madia_et_al_ID_251780404_R1_CLEAN_COPY.docx [file IENZ_A_2496782_SM8241.docx]

Supplemental materials for

Exploring Structure-Activity Relationships of Pyrrolyl Diketo Acid Derivatives as Non-Nucleoside Inhibitors of Terminal Deoxynucleotidyl Transferase Enzyme

Valentina Noemi Madia^1‡^, Nadia Garibaldi^2‡^, Davide Ialongo^1^, Elisa Patacchini^1^, Valeria Tudino^3^, Giuseppe Ruggieri^4,1^, Laura Zarbo^1^, Emanuele Cara^1^, Antonio Coluccia^1^, Marco Artico^5^, Luigi Scipione^1^, Antonella Messore^6^, Francesco Saccoliti^6^, Elisa Mentegari^2^, Giovanni Maga^2^, Roberto Di Santo^1*^, Emmanuele Crespan^2§^ and Roberta Costi^1§^

^1^ Istituto Pasteur-Fondazione Cenci Bolognetti, Dipartimento di Chimica e Tecnologie del Farmaco, “Sapienza” Università di Roma, p.le Aldo Moro 5, I-00185 Rome, Italy; valentinanoemi.madia@uniroma1.it (V.N.M.); davide.ialongo@uniroma1.it (D.I.); elisa.patacchini@uniroma1.it (E.P.); laura.zarbo@uniroma1.it (L.Z.); emanuele.cara@uniroma1.it (E.Ca.); antonio.coluccia@uniroma1.it (A.C.); luigi.scipione@uniroma1.it (L.S.); roberto.disanto@uniroma1.it (R.D.S.); roberta.costi@uniroma1.it (R.C.).

^2^ Institute of Molecular Genetics IGM-CNR, via Abbiategrasso 207, I-27100 Pavia, Italy; nadia.garibaldi@unipv.it (N. G.); elisa.mentegari@igm.cnr.it (E. M.); emmanuele.crespan@igm.cnr.it (E.Cr.); giovanni.maga@igm.cnr.it (G.M.).

^3^ Department of Biotechnology, Chemistry and Pharmacy, University of Siena, 53100 Siena, Italy; valeria.tudino@unisi.it (V.T.).

^4^ Dottorato di Interesse Nazionale in One Health approaches to infectious diseases and life science research, Dipartimento di Sanità Pubblica, Medicina Sperimentale e Forense, Università degli Studi di Pavia, 27100, Pavia, Italy: giuseppe.ruggieri01@univeristadipavia.it (G.R.).

^5^ Department of Sense Organs, Faculty of Medicine and Odontology, “Sapienza” Università di Roma, p.le Aldo Moro 5, I-00185 Rome, Italy; marco.artico@uniroma1.it (M.A.).

^6^ Department of Life Science, Health, and Health Professions, Link Campus University, Via del Casale di San Pio V 44, I-00165, Rome, Italy; a.messore@unilink.it (A.M.); f.saccoliti@unilink.it (F.S.).

***** Correspondence: roberto.disanto@uniroma1.it; Tel.: +39 0649913150.

**‡** These authors contributed equally to this work.

**§** These authors should be considered joint senior authors.

**Contents:**

| 1. Specific Procedures and Characterization | 2-19 |
| --- | --- |
| 1. Table S1 | 20-21 |
| 1. Figure S1 | 22 |
| 1. Figure S2 | 23 |
| 1. References | 24-25 |
| 1. Figure S3 – S50: ^1^H NMR and FTIR Spectra for compounds **5c-k,m**, **6c-k,m** **7c-d**, **8c-d** | 26-73 |

**Specific Procedures and Characterization**

**Ethyl (2*Z*,5*E*)-6-(1-(4-fluorobenzyl)-1*H*-pyrrol-2-yl)-2-hydroxy-4-oxohexa-2,5-dienoate (5a).** Synthesis, analytical and spectroscopic data are reported in literature^1^.

**Ethyl (2*Z*,5*E*)-6-(1-(4-fluorobenzyl)-4-phenyl-1*H*-pyrrol-2-yl)-2-hydroxy-4-oxohexa-2,5-dienoate (5b).** Synthesis, analytical and spectroscopic data are reported in literature^2^.

**(2*Z*,5*E*)-ethyl 6-(4-(2,3-dihydrobenzo[*b*][1,4]dioxine-6-carbonyl)-1-(4-fluorobenzyl)-1*H*-pyrrol-2-yl)-2-hydroxy-4-oxohexa-2,5-dienoate (5c).** Compound **5c** was prepared from **15c** by means GP D. 73%; 162 °C; IR (cm^-1^) 3396 (OH), 1725 (CO ester), 1610 (CO ketone); ^1^H NMR (DMSO-*d*_6_, 400 MHz) *δ* (ppm) 1.12 (t, 3H, *J* = 8 Hz, CH_2_CH_3_), 4.20 (m, 6H, *CH_2_*CH_3_ and CH_2_), 5.42 (s, 2H, CH*_2_*), 6.39 (s, 1H, hexenoate C3-H), 6.91 (d, 2H, *J_o_* = 8 Hz, benzene H), 7.08-7.16 (m, 4H, *J_t_* = 16 Hz, hexenoate C5-H, benzene H and CH pyrrole), 7.24 (s, 1H, benzene H), 7.29-7.31 (m, 2H, benzene H), 7.49 (d, 1H, *J_t_* = 16 Hz, hexenoate C6-H), 7.87 (s, 1H, CH pyrrole). Anal. Calcd for C_28_H_24_FNO_7_: C, 66.53; H, 4.79; F, 3.76; N, 2.77%. Found: C, 66.52; H, 4.78; F, 3.76; N, 2.77%.

**(2*Z*,5*E*)-ethyl 6-(1-(4-fluorobenzyl)-4-(4-(naphthalen-2-ylmethoxy)benzoyl)-1*H*-pyrrol-2-yl)-2-hydroxy-4-oxohexa-2,5-dienoate (5d).** Compound **5d** was prepared from **15d** by means GP D 70%; 157 °C; IR (cm^-1^) 3055 (OH), 1732 (CO ester), 1618 (CO ketone); ^1^H NMR (DMSO-*d*_6_, 400 MHz) *δ* (ppm) 1.27 (t, 3H, *J* = 8 Hz, CH_2_*CH_3_*), 4.27 (q, 2H, *J* = 8 Hz *CH_2_*CH_3_), 5.41 (s, 2H, OCH_2_), 5.50 (s, 2H, CH_2_), 6.48 (s, 1H, hexenoate C3-H), 7.16-7.26 (m, 6H, CH pyrrole, hexenoate C5-H and benzene H), 7.52-7.55 (m, 2H, benzene H), 7.61-7.63 (m, 2H, benzene H), 7.81-7.88 (m, 3H, hexenoate C6-H and benzene H), 7.93-7.98 (m, 5H, benzene H), 8.03 (s, 1H, CH pyrrole). Anal. Calcd for C_37_H_30_FNO_6_: C, 73.62; H, 5.01; F, 3.15; N, 2.32%. Found C, 73.60; H, 5.01; F, 3.14; N, 2.32%.

**(*E*)-ethyl 6-(1-(4-fluorobenzyl)-4-(4-phenoxybenzoyl)-1*H*-pyrrol-2-yl)-2,4-dioxohex-5-enoate (5e).** Compound **5e** was prepared from **15e** by means GP D. 75%; 160 °C; IR (cm^-1^) 3400 (OH), 1727 (CO ester), 1584 (CO ketone); ^1^H NMR (DMSO-*d*_6_, 400 MHz) *δ* (ppm) 1.10 (t, 3H, *J* = 8 Hz, CH_2_*CH_3_*), 4.00 (q, 2H, *J* = 8 Hz, *CH_2_*CH_3_), 5.36 (s, 2H, CH_2_), 6.04 (s, 1H, hexenoate C3-H), 6.98 (d, 1H, *J_o_* = 8 Hz, benzene H), 7.06-7.18 (m, 12H, CH pyrrole, hexenoate C5-H, hexenoate C6-H and benzene H), 7.37 (t, 2H, *J_o_* = 8 Hz, benzene H), 7.78-7.80 (m, 2H, benzene H and CH pyrrole). Anal. Calcd for C_32_H_26_FNO_6_: C, 71.23; H, 4.86; F, 3.52; N, 2.60%. Found C, 71.21; H, 4.90; F, 3.51; N, 2.60%.

**(2*E*,5*E*)-ethyl 6-(1-(4-fluorobenzyl)-4-(4-nitrobenzoyl)-1*H*-pyrrol-2-yl)-2-hydroxy-4-oxohexa-2,5-dienoate (5f).** Compound **5f** was prepared from **15f** by means GP D. 73%; 155 °C; IR (cm^-1^) 3108 (OH), 1731 (CO ester), 1598 (CO ketone); ^1^H NMR (DMSO-*d*_6_, 400 MHz) *δ* (ppm) 1.26 (t, 3H, *J* = 8 Hz, CH_2_*CH_3_*), 4.27 (q, 2H, *J* = 8 Hz, *CH_2_*CH_3_), 5.52 (s, 2H, CH_2_), 6.50 (s, 1H, hexenoate C3-H), 6.96 (d, 1H, *J_t_* = 16 Hz, hexenoate C5-H), 7.16-7.27 (m, 4H, benzene H), 7.45 (s, 1H, CH pyrrole), 7.61 (d, 2H, *J_t_* = 16 Hz, hexenoate C6-H), 8.03-8.06 (m, 3H, CH pyrrole and benzene H), 8.37 (d, 2H, *J_o_* = 8 Hz, benzene H). Anal. Calcd for C_26_H_21_FN_2_O_7_: C, 63.41; H, 4.30; F, 3.86; N, 5.69%. Found C, 63.38; H, 4.28; F, 3.36; N, 5.68%.

**(2*Z*,5*E*)-ethyl 6-(4-(4-(3-bromo-4-methoxybenzamido)benzoyl)-1-(4-fluorobenzyl)-1*H*-pyrrol-2-yl)-2-hydroxy-4-oxohexa-2,5-dienoate (5g).** Compound **5g** was prepared from **15g** by means GP D. 68%; 175 °C; IR (cm^-1^) 2365 (OH), 1725 (CO ester), 1620 (CO ketone); ^1^H NMR (DMSO-*d*_6_, 400 MHz) *δ* (ppm) 1.29 (t, 3H, *J* = 8 Hz, CH_2_*CH_3_*), 3.96 (s, 3H, OCH_3_), 4.27 (q, 2H, *J* = 8 Hz, *CH_2_*CH_3_), 5.50 (s, 2H, CH_2_), 6.50 (s, 1H, hexenoate C3-H), 6.95 (d, 1H, *J_t_* = 16 Hz, hexenoate C5-H), 7.19-7.31 (m, 5H, benzene H), 7.43 (s, 1H, CH pyrrole), 7.62 (d, 1H, *J_t_* = 16 Hz, hexenoate C6-H), 7.83-8.06 (m, 6H, benzene H), 8.27 (s, 1H, CH pyrrole), 10.50 (s, 1H, OH). Anal. Calcd for C_34_H_28_BrFN_2_O_7_: C, 60.45; H, 4.18; Br, 11.83; F, 2.81; N, 4.15%. Found: C, 60.42; H, 4.17; Br, 11.89; F, 2.81; N, 4.10%.

**(2*Z*,5*E*)-ethyl 6-(4-benzoyl-1-(4-chlorobenzyl)-1*H*-pyrrol-2-yl)-2-hydroxy-4-oxohexa-2,5-dienoate (5h).** Compound **5h** was prepared from **15h** by means GP D. 73%; 152 °C; IR (cm^-1^) 2365 (OH), 1730 (CO ester), 1620 (CO ketone); ^1^H NMR (DMSO-*d*_6_, 400 MHz) *δ* (ppm) 1.32 (t, 3H, CH_2_*CH_3_*), 4.29 (q, 2H, *CH_2_*CH_3_), 5.57 (s, 2H, CH_2_), 6.50 (s, 1H, hexenoate C3-H), 7.03 (d, 1H, *J_t_* = 16 Hz, hexenoate C5-H), 7.23 (d, 2H, *J*_o_= 8 Hz benzene H), 7.40 (s, 1H, CH pyrrole), 7.46-7.48 (m, 2H, benzene H), 7.58-7.61 (m, 3H, hexenoate C6-H and benzene H), 7.68 (t, 1H, *J*_o_= 8 Hz, benzene H), 7.87 (d, 2H, *J*_o_= 8 Hz benzene H), 8.02 (s, 1H, CH pyrrole). Anal. Calcd for C_26_H_22_ClNO_5_: C, 67.32; H, 4.78; Cl, 7.64; N, 3.02%. Found C, 67.30; H, 4.77; Cl, 7.62; N, 3.02%.

**(2*Z*,5*E*)-ethyl 6-(4-benzoyl-1-(4-methoxybenzyl)-1*H*-pyrrol-2-yl)-2-hydroxy-4-oxohexa-2,5-dienoate (5i).** Compound **5i** was prepared from **15i** by means GP D. 70%; 155 °C; IR (cm^-1^) 2365 (OH), 1730 (CO ester), 1640 (CO ketone), 1230 (C-O-C), 1041 (C-O-C); ^1^H NMR (DMSO-*d*_6_, 400 MHz) *δ* (ppm) 1.29 (t, 3H, *J* = 8 Hz, CH_2_*CH_3_*), 3.73 (s, 3H, OCH_3_), 4.23 (q, 2H, *J* = 8 Hz, *CH_2_*CH_3_), 5.40 (s, 2H, CH_2_), 6.48 (s, 1H, hexenoate C3-H), 6.96-6.98 (m, 3H, CH pyrrole and benzene H), 7.16-7.21 (m, 3H, hexenoate C5-H and benzene H), 7.58-7.71 (m, 4H, hexenoate C6-H and benzene H), 7.85 (d, 2H, *J_o_* = 8 Hz benzene H), 7.90 (s, 1H, CH pyrrole). Anal. Calcd for C_27_H_25_FNO_6_: C, 70.58; H, 5.48; N, 3.05%. Found C, 70.55; H, 5.46; N, 3.05%.

**Ethyl (2*Z*,5*E*)-6-(4-benzoyl-1-(naphthalen-1-ylmethyl)-1*H*-pyrrol-2-yl)-2-hydroxy-4-oxohexa-2,5-dienoate (5j).** Compound **5j** was prepared from **15j** by means GP D. 73%; 154 °C; IR (cm^-1^) 2367 (OH), 1723 (CO ester), 1621 (CO ketone); ^1^H NMR (DMSO-*d*_6_, 400 MHz) *δ* (ppm) 1.24 (t, 3H, *J* = 8 Hz, CH_2_*CH_3_*), 4.24 (q, 2H, *J* = 8 Hz, *CH_2_*CH_3_), 5.69 (s, 2H, CH_2_), 6.40 (s, 1H, hexenoate C3-H), 6.89 (d, 1H, *J_t_* = 16 Hz, hexenoate C5-H), 7.34 (d, 2H, *J_O_* = 8 Hz, benzene H), 7.44-7.68 (m, 9H, CH pyrrole and benzene H), 7.85-7.94 (m, 4H, hexenoate C6-H and benzene H), 8.12 (s, 1H,CH pyrrole). Anal. Calcd for C_30_H_25_NO_5_: C, 75.14; H, 5.26; N, 2.92%. Found C, 75.10; H, 5.24; N, 2.92%.

**(2*Z*,5*E*)-ethyl 2-hydroxy-4-oxo-6-(4-(3,4,5-trimethoxybenzoyl)-1*H*-pyrrol-2-yl)hexa-2,5-dienoate (5k).** Compound **5k** was prepared from **15k** by means GP D. 68%; 161 °C; IR (cm^-1^) 3251 and 2971 (NH and OH), 1725 (CO ester), 1630 (CO ketone); ^1^H NMR (DMSO-*d*_6_, 400 MHz) *δ* (ppm) 1.28 (t, 3H, *J* = 8 Hz, CH_2_*CH_3_*), 3.77 (s, 3H, OCH_3_), 3.83 (s, 6H, OCH_3_), 4.26 (q, 2H, *J* = 8 Hz, *CH_2_*CH_3_), 6.48 (s, 1H, hexenoate C3-H), 6.81 (d, 1H, *J_t_* = 16 Hz, hexenoate C5-H), 7.08 (s, 2H, benzene H), 7.24 (s, 1H, CH pyrrole), 7.70-7.74 (d, 1H, J *J_t_* = 16 Hz, hexenoate C6-H), 7.87-7.88 (m, 1H, CH pyrrole), 12.41 (s, 1H, NH). Anal. Calcd for C_22_H_23_NO_8_: C, 61.53; H, 5.40; N, 3.26%. Found: C, 61.50; H, 5.39; N, 3.26%.

**(2*Z*,5*E*)- ethyl 6-(4-benzoyl-1*H*-pyrrol-2-yl)-2-hydroxy-4-oxohexa-2,5-dienoate (5l).** Synthesis, analytical and spectroscopic data are reported in literature^3^.

**Ethyl (2*Z*,5*E*)-6-(4-benzoyl-1-((4-fluorophenyl)sulfonyl)-1*H*-pyrrol-2-yl)-2-hydroxy-4-oxohexa-2,5-dienoate (5m).** Compound **5m** was prepared from **15m** by means GP D. 73%; 157 °C; IR (cm^-1^) 3261 (OH), 1720 (CO ester), 1622 (CO ketone), 1356 and 1120 (SO_2_); ^1^H NMR (DMSO-*d*_6_, 400 MHz) *δ* (ppm) 1.30 (t, 3H, *J* = 8 Hz, CH_2_*CH_3_*), 4.29 (q, 2H, *J* = 8 Hz, *CH_2_*CH_3_), 6.49 (s, 1H, hexenoate C3-H), 6.83 (d, 1H, *J_t_* = 16 Hz, hexenoate C5-H), 7.20 (s, 1H, CH pyrrole), 7.53-7.81 (m, 11H, CH pyrrole, hexenoate C6-H and benzene H), 12.43 (s, 1H, OH). Anal. Calcd for C_25_H_20_FNO_7_S: C, 60.36; H, 4.05; F, 3.82; N, 2.82; S, 6.45%. Found C, 60.30 H, 4.03; F, 3.81; N, 2.82; S, 6.44%.

**(2*Z*,5*E*)-6-(1-(4-fluorobenzyl)-1*H*-pyrrol-2-yl)-2-hydroxy-4-oxohexa-2,5-dienoic acid (6a).** Synthesis, analytical and spectroscopic data are reported in literature^1^.

**(2*Z*,5*E*)-6-(1-(4-fluorobenzyl)-4-phenyl-1*H*-pyrrol-2-yl)-2-hydroxy-4-oxohexa-2,5-dienoic acid (6b).** Synthesis, analytical and spectroscopic data are reported in literature^2^.

**(2*Z*,5*E*)-6-(4-(2,3-dihydrobenzo[*b*][1,4]dioxine-6-carbonyl)-1-(4-fluorobenzyl)-1*H*-pyrrol-2-yl)-2-hydroxy-4-oxohexa-2,5-dienoic acid (6c).** Compound **6c** was prepared from **5c** by means GP E. 88%; 182 °C; IR (cm^-1^) 3129 (OH), 1730 (CO acid), 1606 (CO ketone); ^1^H NMR (DMSO-*d*_6_, 400 MHz) *δ* (ppm) 4.22-4.24 (m, 4H, CH_2_), 5.41 (s, 2H, CH_2_), 6.41 (s, 1H, hexenoate C3-H), 6.92 (d, 2H, *J_o_* = 8 Hz, benzene H) 7.08-7.16 (m, 4H, hexenoate C5-H, benzene H and CH pyrrole), 7.24-7.31 (m, 2H, benzene H), 7.38 (m, 3H, benzene H), 7.50 (d, 1H, *J_t_* = 16 Hz, hexenoate C6-H), 7.86 (s, 1H, CH pyrrole). Anal. Calcd for C_26_H_20_FNO_7_: C, 65.41; H, 4.22; F, 3.98; N, 2.93%. Found: C, 65.38; H, 4.21; F, 3.97; N, 2.93%.

**(2*Z*,5*E*)-6-(1-(4-fluorobenzyl)-4-(4-(naphthalen-2-ylmethoxy)benzoyl)-1*H*-pyrrol-2-yl)-2-hydroxy-4-oxohexa-2,5-dienoic acid (6d).** Compound **6d** was prepared from **5d** by means GP E. 85%; 180 °C; IR (cm^-1^) 3132 (NH and OH), 1719 (CO acid), 1591 (CO ketone); ^1^H NMR (DMSO-*d*_6_, 400 MHz) *δ* (ppm) 5.41 (s, 2H, OCH_2_), 5.49 (s, 2H, CH_2_), 6.40 (s, 1H, hexenoate C3-H), 6.86 (d, 1H, *J_t_* = 16 Hz, hexenoate C5-H), 7.17-7.24 (m, 4H, benzene H), 7.37 (s, 1H, CH pyrrole), 7.53-7.56 (m, 3H, hexenoate C6-H and benzene H), 7.62 (d, 2H, *J_O_* = 8 Hz, benzene H), 7.86 (d, 2H, *J_o_* = 8 Hz, benzene H), 7.93-7.99 (m, 5H, benzene H), 8.03 (s, 1H, CH pyrrole). Anal. Calcd for C_35_H_26_FNO_6_: C, 73.03; H, 4.55; F, 3.30; N, 2.43%. Found C, 72.95; H, 4.53; F, 3.29; N, 2.43%.

**(*E*)-6-(1-(4-fluorobenzyl)-4-(4-phenoxybenzoyl)-1*H*-pyrrol-2-yl)-2,4-dioxohex-5-enoic acid (6e).**

Compound **6e** was prepared from **5e** by means GP E. 90%; 181 °C; IR (cm^-1^) 3131 (OH), 1730 (CO acid), 1582 (CO ketone); ^1^H NMR (DMSO-*d*_6_, 400 MHz) *δ* (ppm) 5.41 (s, 2H, CH_2_), 6.04 (s, 1H, hexenoate C3-H), 6.78 (d, 1H, *J_t_* = 16 Hz, hexenoate C5-H), 6.99 (d, 2H, *J_O_* = 8 Hz, benzene H), 7.01-7-19 (m, 7H, CH pyrrole and benzene H), 7.27 (d, 1H, *J_t_* = 16 Hz, hexenoate C6-H), 7.34-7.46 (m, 3H, benzene H), 7.79 (d, 2H, *J_O_* = 8 Hz, benzene H), 7.88 (s, 1H, CH pyrrole), 14.22 (s, 1H, OH). Anal. Calcd for C_30_H_22_FNO_6_: C, 70.45; H, 4.34; F, 3.71; N, 2.74%. Found C, 70.40; H, 4.33; F, 3.71; N, 2.73%.

**(2*E*,5*E*)-6-(1-(4-fluorobenzyl)-4-(4-nitrobenzoyl)-1*H*-pyrrol-2-yl)-2-hydroxy-4-oxohexa-2,5-dienoic acid (6f).** Compound **6f** was prepared from **5f** by means GP E. 90%; 185 °C; IR (cm^-1^) 3675 (OH), 1686 (CO acid), 1599 (CO ketone); ^1^H NMR (DMSO-*d*_6_, 400 MHz) *δ* (ppm) 5.51 (s, 2H, CH_2_), 6.47 (s, 1H, hexenoate C3-H), 6.92 (d, 1H, *J_t_* = 16 Hz, hexenoate C5-H), 7.16-7.27 (m, 4H, benzene H), 7.37 (d, 2H, *J_O_* = 8 Hz, benzene H), 7.43 (s, 1H, CH pyrrole), 7.58 (d, 1H, *J_t_* = 16 Hz, hexenoate C6-H), 8.02-8.06 (m, 3H, CH pyrrole and benzene H), 14.0 (s, 1H, COOH). Anal. Calcd for C_24_H_17_FN_2_O_7_: C, 62.07; H, 3.69; F, 4.09; N, 6.03%. Found C, 62.03; H, 3.69; F, 4.05; N, 6.01%.

**(2*Z*,5*E*)-6-(4-(4-(3-bromo-4-methoxybenzamido)benzoyl)-1-(4-fluorobenzyl)-1*H*-pyrrol-2-yl)-2-hydroxy-4-oxohexa-2,5-dienoic acid (6g).** Compound **6g** was prepared from **5g** by means GP E. 85%; 188 °C; IR (cm^-1^) 3109 (NH and OH), 1730 (CO acid), 1596 (CO ketone); ^1^H NMR (DMSO-*d*_6_, 400 MHz) *δ* (ppm) 3.96 (s, 3H, OCH_3_), 5.52 (s, 2H, CH_2_) 6.49 (s, 1H, hexenoate C3-H), 6.91 (d, 1H, *J_t_* = 16 Hz, hexenoate C5-H), 7.17-7.28 (m, 6H, benzene H and NH), 7.43 (br s, 1H, CH pyrrole), 7.60 (d, 1H, *J_t_* = 16 Hz, hexenoate C6-H), 7.86-8.06 (m, 6H, benzene H), 8.27 (s, 1H, CH pyrrole), 10.45 (s, 1H, OH), 14.01 (s, 1H, *COOH*). Anal. Calcd for C_32_H_24_BrFN_2_O_7_: C, 59.36; H, 3.74; Br, 12.34; F, 2.93; N, 4.33%. Found: C, 59.33; H, 3.72; Br, 12.31; F, 2.93; N, 4.32%.

**(2*Z*,5*E*)-6-(4-benzoyl-1-(4-chlorobenzyl)-1*H*-pyrrol-2-yl)-2-hydroxy-4-oxohexa-2,5-dienoic acid (6h).** Compound **6h** was prepared from **5h** by means GP E. 98%; 177 °C; IR (cm^-1^) 3199 (OH), 1731 (CO acid), 1620 (CO ketone); ^1^H NMR (DMSO-*d*_6_, 400 MHz) *δ* (ppm) 5.57 (s, 2H, CH_2_), 6.51 (s, 1H, hexenoate C3-H), 6.97 (d, 1H, *J_t_* = 16 Hz, hexenoate C5-H), 7.23 (d, 2H, *J_o_* = 8 Hz, benzene H), 7.46-7.48 (m, 3H, CH pyrrole and benzene H), 7.58-7.61 (m, 4H, hexenoate C6-H and benzene H), 7.87 (d 2H, *J_o_* = 8 Hz, benzene H), 8.02 (s, 1H, CH pyrrole). Anal. Calcd for C_24_H_18_ClNO_5_: C, 66.14; H, 4.16; Cl, 8.13; N, 3.21%. Found C, 66.11; H, 4.15; Cl, 8.12; N, 3.20%.

**(2*Z*,5*E*)-6-(4-benzoyl-1-(4-methoxybenzyl)-1*H*-pyrrol-2-yl)-2-hydroxy-4-oxohexa-2,5-dienoic acid (6i).** Compound **6i** was prepared from **5i** by means GP E. 95%; 180 °C; IR (cm^-1^) 3199 (OH), 1731 (CO acid), 1610 (CO ketone); ^1^H NMR (DMSO-*d*_6_, 400 MHz) *δ* (ppm) 3.72 (s, 3H, OCH_3_), 5.43 (s, 2H, CH_2_), 6.48 (s, 1H, hexenoate C3-H), 6.89-6.93 (m, 3H, hexenoate C5-H and benzene H), 7.14 (d, 2H, *J_o_* = 8 Hz, benzene H), 7.39 (s, 1H, CH pyrrole), 7.54-7.57 (m, 2H, benzene H), 7.62-7.64 (m, 2H, hexenoate C6-H and benzene H), 7.80 (d, 2H, *J_o_* = 8 Hz, benzene H), 7.94 (s, 1H, CH pyrrole). Anal. Calcd for C_25_H_21_NO_6_: C, 69.60; H, 4.91; N, 3.25%. Found C, 69.56; H, 4.90; N, 3.24%.

**(2*Z*,5*E*)-6-(4-benzoyl-1-(naphthalen-1-ylmethyl)-1*H*-pyrrol-2-yl)-2-hydroxy-4-oxohexa-2,5-dienoic acid (6j).** Compound **6j** was prepared from **5j** by means GP E. 90%; 183 °C; IR (cm^-1^) 2923 (OH), 1724 (CO acid), 1597 (CO ketone); ^1^H NMR (DMSO-*d*_6_, 400 MHz) *δ* (ppm) 5.67 (s, 2H, CH_2_), 6.44 (s, 1H, hexenoate C3-H), 6.89 (d, 1H, *J_t_* = 16 Hz, hexenoate C5-H), 7.32-7.68 (m, 10H, CH pyrrole and benzene H), 7.83-7.90 (m, 6H, benzene H), 7.94 (s, 1H, CH pyrrole). Anal. Calcd for C_28_H_21_NO_5_: C, 74.49; H, 4.69; N, 3.10%. Found C, 74.46; H, 4.68; N, 3.09%.

**(2*Z*,5*E*)-2-hydroxy-4-oxo-6-(4-(3,4,5-trimethoxybenzoyl)-1*H*-pyrrol-2-yl)hexa-2,5-dienoic acid (6k).** Compound **6k** was prepared from **5k** by means GP E. 95%; 185 °C; IR (cm^-1^) 3213 (NH and OH), 1731 (CO acid), 1571 (CO ketone); ^1^H NMR (DMSO-*d*_6_, 400 MHz) *δ* (ppm) 3.76 (s, 3H, OCH_3_), 3.84 (s, 6H, OCH_3_), 6.46 (s, 1H, hexenoate C3-H), 6.86 (d, 1H, *J_t_* = 16 Hz, hexenoate C5-H), 7.08 (s, 2H, benzene H), 7.21 (s, 1H, CH pyrrole), 7.68 (d, 1H, *J_t_* =16 Hz, hexenoate C6-H), 7.82 (bs, 1H, CH pyrrole), 12.59 (s, 1H, NH). Anal. Calcd for C_20_H_19_NO_8_: C, 59.85; H, 4.77; N, 3.49%. Found: C, 59.82; H, 4.76; N, 3.49%.

**(2*Z*,5*E*)-6-(4-benzoyl-1*H*-pyrrol-2-yl)-2-hydroxy-4-oxohexa-2,5-dienoic acid (6l).** Synthesis, analytical and spectroscopic data are reported in literature^3^.

**(2*Z*,5*E*)-6-(4-benzoyl-1-((4-fluorophenyl)sulfonyl)-1*H*-pyrrol-2-yl)-2-hydroxy-4-oxohexa-2,5-dienoic acid (6m).** Compound **6m** was prepared from **5m** by means GP E. 90%; 175 °C; IR (cm^-1^) 3226 (OH), 1727 (CO acid), 1647 (CO ketone), 1383 and 1152 (SO_2_); ^1^H NMR (DMSO-*d*_6_, 400 MHz) *δ* (ppm) 6.48 (s, 1H, hexenoate C3-H), 6.82 (d, 1H, *J_t_* = 16 Hz, hexenoate C5-H), 7.18 (s, 1H, CH pyrrole), 7.53-7.81 (m, 11H, CH pyrrole, hexenoate C6-H and benzene H), 12.43 (s, 1H, OH), 14.0 (s, 1H, COOH). Anal. Calcd for C_23_H_16_FNO_7_S: C, 58.85; H, 3.44; F, 4.05; N, 2.98; S, 6.83%. Found C, 58.81; H, 3.44; F, 4.04; N, 2.98; S, 6.82%.

**Ethyl (2*Z*,5*E*)-6-(1-(4-fluorobenzyl)-1*H*-pyrrol-3-yl)-2-hydroxy-4-oxohexa-2,5-dienoate (7a).** Synthesis, analytical and spectroscopic data are reported in literature^3^.

**Ethyl (2*Z*,5*E*)-6-(1-(4-fluorobenzyl)-4-phenyl-1*H*-pyrrol-3-yl)-2-hydroxy-4-oxohexa-2,5-dienoate (7b).** Synthesis, analytical and spectroscopic data are reported in literature^2^.

**Ethyl (2*Z*,5*E*)-2-hydroxy-4-oxo-6-(4-phenyl-1*H*-pyrrol-3-yl)hexa-2,5-dienoate (7c).** Compound **7c** was prepared from (*E*)-4-(4-phenyl-1*H*-pyrrol-3-yl)but-3-en-2-one^1^ by means of GP D. 70%; 180 °C; IR (cm^-1^) 1735 (CO ester), 1681 (CO ketone); ^1^H NMR (DMSO-*d*_6_, 400 MHz) *δ* (ppm) 1.31 (t, 3H, *J* = 8 Hz, CH_2_*CH_3_*), 4.30 (q, 2H, *J* = 8 Hz, *CH_2_*CH_3_), 6.88 (s, 1H, hexenoate C3-H), 6.97-7.01 (d, 1H, *J_t_* =16 Hz, hexenoate C5-H), 7.24 (t, 1H, *J_o_* = 8 Hz, benzene H), 7.34-7.39 (m, 3H, CH pyrrole and benzene H), 7.46-7.48 (m, 2H, benzene H), 7.73 (d, 1H, *J_t_* = 16 Hz, hexenoate C6-H), 8.05 (s, 1H, CH pyrrole), 12.00 (s, 1H, OH). Anal. Calcd For C_18_H_17_NO_4_: C, 69.44; H, 5.50; N, 4.50%. Found: C, 69.41; H, 5.49, N, 4.49%.

**Ethyl (2*Z*,5*E*)-2-hydroxy-4-oxo-6-(4-(3,4,5-trimethoxyphenyl)-1*H*-pyrrol-3-yl)hexa-2,5-dienoate (7d).** Compound **7d** was prepared from **17** by means of GP D. 75%; 188 °C; IR (cm^-1^) 1733 (CO ester), 1685 (CO ketone), 1250 (C-O-C), 1048 (C-O-C); ^1^H NMR (DMSO-*d*_6_, 400 MHz) *δ* (ppm) 1.29 (t, 3H, *J* = 8 Hz, CH_2_*CH_3_*), 3.66 (s, 3H, OCH_3_), 3.81 (s, 6H, OCH_3_), 4.27 (q, 2H, *J* = 8 Hz, *CH_2_*CH_3_), 6.76 (s, 2H, benzene H), 6.88 (s, 1H, hexenoate C3-H), 6.91 (d, 1H, *J_t_* = 16 Hz, hexenoate C5-H), 7.33 (s, 1H, CH pyrrole), 7.57 (d, 1H, *J_t_* = 16 Hz, hexenoate C6-H), 8.04 (s, 1H, CH pyrrole), 11.96 (s, 1H, OH). Anal. Calcd For C_21_H_23_NO_7_: C, 62.84; H, 5.78; N, 3.49%. Found: C, 62.80; H, 5.77; N, 3.48.

**(2*Z*,5*E*)-6-(1-(4-fluorobenzyl)-1*H*-pyrrol-3-yl)-2-hydroxy-4-oxohexa-2,5-dienoic acid (8a).** Synthesis, analytical and spectroscopic data are reported in literature^3^.

**(2*Z*,5*E*)-6-(1-(4-fluorobenzyl)-4-phenyl-1*H*-pyrrol-3-yl)-2-hydroxy-4-oxohexa-2,5-dienoic acid (8b).** Synthesis, analytical and spectroscopic data are reported in literature^2^.

**(2*Z*,5*E*)-2-hydroxy-4-oxo-6-(4-phenyl-1*H*-pyrrol-3-yl)hexa-2,5-dienoic acid (8c).** Compound **8c** was prepared from **7c** by means of GP E. 90%; 181 °C; IR (cm^-1^) 3228 (OH), 1715 (CO acid); ^1^H NMR (DMSO-*d*_6_, 400 MHz) *δ* (ppm) 6.86 (s, 1H, hexenoate C3-H), 6.97 (d, 1H, *J_t_* = 16 Hz, hexenoate C5-H), 7.23 (t, 1H, *J* = 8 Hz, benzene H), 7.32-7.39 (m, 3H*,* CH pyrrole and benzene H), 7.42-7.48 (m, 2H, benzene H), 7.72-7.76 (d, 1H, *J_t_* = 16 Hz, hexenoate C6-H), 8.01-8.02 (m, 1H, CH pyrrole). Anal. Calcd For C_16_H_13_NO_4_: C, 67.84; H, 4.63; N, 4.94%. Found: C, 67.80; H, 4.62, N, 4.93%.

**(2*Z*,5*E*)-2-hydroxy-4-oxo-6-(4-(3,4,5-trimethoxyphenyl)-1*H*-pyrrol-3-yl)hexa-2,5-dienoic acid (8d).** Compound **8d** was prepared from **7d** by means of GP E. 90%; 180-182 °C; IR (cm^-1^) 3200 (OH), 1710 (CO acid), 1255 (C-O-C), 1046 (C-O-C); ^1^H NMR (DMSO-*d*_6_, 400 MHz) *δ* (ppm) 3.66 (s, 3H, OCH_3_), 3.81 (s, 6H, OCH_3_), 6.72 (s, 2H, benzene H), 6.86 (s, 1H, hexenoate C3-H), 6.91 (d, 1H, *J_t_* = 16 Hz, hexenoate C5-H), 7.32 (s, 1H, CH pyrrole), 7.58 (d, 1H, *J_t_* = 16 Hz, hexenoate C6-H), 7.99 (s, 1H, CH pyrrole), 11.91 (s, 1H, OH), 13.77 (s, 1H, COOH). Anal. Calcd For C_19_H_19_NO_7_: C, 61.12; H, 5.13; N, 3.75%. Found: C, 61.07; H, 5.11; N, 3.74%.

**Ethyl (*Z*)-4-(1-(4-fluorobenzyl)-1*H*-pyrrol-2-yl)-2-hydroxy-4-oxobut-2-enoate (9a).** Synthesis, analytical and spectroscopic data are reported in literature^2^.

**Ethyl (*Z*)-4-(1-(4-fluorobenzyl)-4-phenyl-1*H*-pyrrol-2-yl)-2-hydroxy-4-oxobut-2-enoate (9b).** Synthesis, analytical and spectroscopic data are reported in literature^2^.

**(*Z*)-4-(1-(4-fluorobenzyl)-1*H*-pyrrol-2-yl)-2-hydroxy-4-oxobut-2-enoic acid (10a).** Synthesis, analytical and spectroscopic data are reported in literature^2^.

**(*Z*)-4-(1-(4-fluorobenzyl)-4-phenyl-1*H*-pyrrol-2-yl)-2-hydroxy-4-oxobut-2-enoic acid (10b).** Synthesis, analytical and spectroscopic data are reported in literature^2^.

**Ethyl (*Z*)-4-(1-(4-fluorobenzyl)-1*H*-pyrrol-3-yl)-2-hydroxy-4-oxobut-2-enoate (11a).** Synthesis, analytical and spectroscopic data are reported in literature^2^.

**Ethyl 4-(1-(4-fluorobenzyl)-4-phenyl-1*H*-pyrrol-3-yl)-2,4-dioxobutanoate (11b).** Synthesis, analytical and spectroscopic data are reported in literature^2^.

**(*Z*)-4-(1-(4-fluorobenzyl)-1*H*-pyrrol-3-yl)-2-hydroxy-4-oxobut-2-enoic acid (12a).** Synthesis, analytical and spectroscopic data are reported in literature^2^.

**4-(1-(4-fluorobenzyl)-4-phenyl-1*H*-pyrrol-3-yl)-2,4-dioxobutanoic acid (12b).** Synthesis, analytical and spectroscopic data are reported in literature^2^.

**4-(2,3-Dihydrobenzo[*b*][1,4]dioxine-6-carbonyl)-1*H*-pyrrole-2-carbaldehyde (13c).** Compound **13c** was prepared from pyrrole by means GP A using 2,3-dihydrobenzo[*b*][1,4]dioxine-6-carbonyl chloride. Silica gel, chloroform/ethyl acetate 5:5; 51%; 167 °C; IR (cm^-1^) 3266 (NH), 1654 (CO aldehyde), 1637 (CO ketone), 1297 (C-O-C); ^1^H NMR (DMSO-*d*_6_, 400 MHz) *δ* (ppm) 4.23-4.25 (m, 4H, CH 1,4-dioxane), 6.92 (d, 1H, *J_o_* = 8 Hz, benzene H), 7.22 (d, 1H, *J_m_* = 2 Hz, benzene H), 7.29 (dd, 1H, *J_o_* = 8 Hz, *J_m_* = 2 Hz, benzene H), 7.34 (s, 1H, CH pyrrole) 7.65 (s, 1H, CH pyrrole), 9.52 (s, 1H, aldehyde CH), 12.71 (bs, 1H, NH). Anal. Calcd for C_14_H_11_NO_4_: C, 65.37; H, 4.31; N, 5.45%. Found C, 65.34; H, 4.31; N, 5.44%.

**4-(4-(Naphthalen-2-ylmethoxy)benzoyl)-1*H*-pyrrole-2-carbaldehyde (13d).** Compound **13d** was prepared from pyrrole by means GP A using 4-(naphthalen-2-ylmethoxy)benzoyl chloride. Silica gel, *n*-hexane/ethyl acetate 3:7; 74%; 150°C; IR (cm^-1^) 1665 (CO aldehyde), 1628 (CO ketone), 1258 (C-O-C ether), 1092 (C-O-C ether); ^1^H NMR (DMSO-*d*_6_, 400 MHz) *δ* (ppm) 5.40 (s, 2H, CH_2_-O), 5.58 (s, 2H, CH_2_), 7.14-7.31 (m, 5H, benzene and naphthalene H), 7.51-7.56 (m, 4H, benzene and naphthalene H), 7.86-7.69 (m, 2H, naphthalene H), 8.02 (bs, 1H, CH pyrrole), 8.15 (bs, 1H, CH pyrrole), 9.62 (s, 1H, aldehyde CH). Anal. Calcd for C_23_H_17_NO_3_: C, 77.73; H, 4.82; N, 3.94;%. Found C, 77.68; H, 4.81; N, 3.94%.

**4-(4-phenoxybenzoyl)-1*H*-pyrrole-2-carbaldehyde (13e).** Compound **13e** was prepared from pyrrole by means GP A using 4-phenoxybenzoyl chloride. Silica gel, *n*-hexane/ethyl acetate 7:3; 70%; IR (cm^-1^) 3249 (NH), 1686 (CO aldehyde), 1664 (CO ketone), 1355 (C-O-C); ^1^H NMR (DMSO d_6_) *δ* 7.00 (d, 2H, *J_o_* = 8.4 Hz, benzene H), 7.07 (d, 2H, *J_o_* = 7.5 Hz, benzene H), 7.17 (t, 1H, *J_o_* = 7.5 Hz, benzene H), 7.37-7.41 (m, 3H, benzene H and CH pyrrole), 7.68 (s, 1H, CH pyrrole), 7.79 (d, 2H, *J_o_* = 8.8 Hz, CH benzene H Ar) 9.53 (s, 1H, aldehyde CH), 12.75 (bs, 1H, NH). Anal. Calcd for C_18_H_13_NO_3_: C, 74.22; H, 4.50; N, 4.81%. Found C, 73.98; H, 4.49; N, 4.80%.

**4-(3,4,5-trimethoxybenzoyl)-1*H*-pyrrole-2-carbaldehyde (13k).** Compound **13k** was prepared from pyrrole by means GP A using 3,4,5-trimethoxybenzoyl chloride. Silica gel, chloroform/ethyl acetate 7:3; 50%; IR (cm^-1^) 3231 (NH), 1638 (CO aldehyde and ketone), 1300 (C-O-C); ^1^H NMR (DMSO-*d*_6_, 400 MHz) *δ* (ppm) 3.66 (s, 3H, OCH_3_), 3.75 (s, 6H, OCH_3_), 7.00 (s, 2H, benzene H), 7.40 (s, 1H, CH pyrrole), 7.74 (s, 1H, CH pyrrole), 9.52 (s, 1H, aldehyde CH), 12.71 (bs, 1H, NH). Anal. Calcd for C_15_H_15_NO_5_: C, 62.28; H, 5.23; N, 4.84%. Found C, 62.25; H, 5.22; N, 4.84%.

**4-(2,3-dihydrobenzo[*b*][1,4]dioxine-6-carbonyl)-1-(4-fluorobenzyl)-1*H*-pyrrole-2-carbaldehyde (14c).** Compound **14c** was prepared by means GP B using **13c** and 4-fluorobenzyl bromide as alkylating agent. 5 h; Silica gel, chloroform/ethyl acetate 9.8:0.2; 60%; 175 °C; IR (cm^-1^) 1671 (CO aldehyde), 1631 (CO ketone); ^1^H NMR (DMSO-*d*_6_, 400 MHz) *δ* (ppm) 4.21-4.26 (m, 4H, CH 1,4-dioxane ), 5.49 (s, 2H, CH_2_), 6.93 (d, 1H, J*_o_* = 8 Hz, benzene H), 7.06-7.10 (m, 2H, benzene H and CH pyrrole), 7.18-7.31 (m, 4H, benzene H), 7.44 (s, 1H, CH pyrrole), 8.04 (s, 1H, CH pyrrole), 9.53 (s, 1H, aldehyde CH). Anal. Calcd for C_21_H_16_FNO_4_: C, 69.04; H, 4.41; F, 5.20; N, 3.83%. Found C, 69.00; H, 4.40; F, 5.18; N, 3.83%.

**1-(4-fluorobenzyl)-4-(4-(naphthalen-2-ylmethoxy)benzoyl)-1*H*-pyrrole-2-carbaldehyde (14d).** Compound **14d** was prepared by means GP B using **13d** and 4-fluorobenzyl bromide as alkylating agent. 5 h; Silica gel, *n*-hexane/ethyl acetate 3:7; 74%; 150 °C; IR (cm^-1^)1665 (CO aldehyde), 1628 (CO ketone), 1258 (C-O-C ether), 1092 (C-O-C ether); ^1^H NMR (DMSO-*d*_6_, 400 MHz) *δ* (ppm) 5.40 (s, 2H, CH_2_-O), 5.58 (s, 2H, CH_2_), 7.14-7.31 (m, 7H, benzene and naphthalene H), 7.51-7.56 (m, 4H, benzene and naphthalene H), 7.86-7.69 (m, 5H, benzene and naphthalene H), 8.02 (bs, 1H, CH pyrrole), 8.15 (bs, 1H, CH pyrrole), 9.62 (s, 1H, aldehyde CH). Anal. Calcd for C_30_H_22_FNO_3_: C, 77.74; H, 4.78; F, 4.10; N, 3.02%. Found C, 77.69; H, 4.77; F, 4.09; N, 3.02%.

**1-(4-fluorobenzyl)-4-(4-phenoxybenzoyl)-1*H*-pyrrole-2-carbaldehyde (14e).** Compound **14e** was prepared by means GP B using **13e** and 4-fluorobenzyl bromide as alkylating agent. 4 h; Silica gel, *n*-hexane/ethyl acetate 7:3; 60%; IR (cm^-1^) 1669 (CO aldehyde), 1626 (CO ketone); ^1^H NMR (DMSO-*d*_6_, 400 MHz) *δ* (ppm) 5.47(s, 2H, CH_2_), 6.99 (d, 2H, *J_o_* = 7.6 Hz, benzene H), 7.05-7.08 (m, 3H, benzene H), 7.16-7.20 (m, 4H, benzene H), 7.38 (t, 2H, *J_o_* = 7.2 Hz, benzene H), 7.45 (s, 1H, CH pyrrole), 7.78 (d, 2H, *J_o_* = 7.2 Hz, benzene H), 8.08 (s, 1H, CH pyrrole), 9.52 (s, 1H, aldehyde CH). Anal. Calcd for C_25_H_18_FNO_3_: C, 75.18; H, 4.54; F, 4.76; N, 3.51%. Found C, 75.15; H, 4.53; F, 4.75; N, 3.40%.

**1-(4-fluorobenzyl)-4-(4-nitrobenzoyl)-1*H*-pyrrole-2-carbaldehyde (14f).** Compound **14f** was prepared by means GP B using 4-(4-nitrobenzoyl)-1*H*-pyrrole-2-carbaldehyde^4^ and 4-fluorobenzyl bromide as alkylating agent. 8 h; Silica gel, *n*-hexane/ethyl acetate 5:5; 70%; 149 °C; IR (cm^-1^) 1671 (CO aldehyde), 1641(CO ketone), 1532 (NO_2_); ^1^H NMR (DMSO-*d*_6_, 400 MHz) *δ* (ppm) 5.58 (s, 2H, CH_2_), 7.15-7.20 (m, 2H, benzene H), 7.28-7.32 (m, 2H, benzene H), 7.59 (s, 1H, CH pyrrole), 8.03-8.06 (m, 2H, benzene H), 8.24 (bs, 1H, CH pyrrole), 8.37-8.40 (m, 2H, benzene H), 9.64 (s, 1H, aldehyde CH). Anal. Calcd for C_19_H_13_FN_2_O_4_: C, 64.77; H, 3.72; F, 5.39; N, 7.95%. Found C, 64.70; H, 3.70; F, 5.38; N, 7.94%.

**Synthesis of 3-bromo-*N*-(4-(1-(4-fluorobenzyl)-5-formyl-1*H*-pyrrole-3-carbonyl)phenyl)-4 methoxybenzamide (14g).** A mixture of 3-bromo-4-methoxybenzoyl chloride in anhydrous CH_2_Cl_2_ (20 mL) was added dropwise to a well-stirred solution of **16** (suitable synthesized, see **Supplemental Material**, 1 mmol) and Et_3_N (2 mmol) in anhydrous CH_2_Cl_2_ (30 mL) at 0 °C under inert atmosphere. The reaction was stirred at room for 15 h. Upon reaction completion, checked by TLC, the mixture was diluted with CH_2_Cl_2_ and washed with water, NaHCO_3_ s.s., 1 N HCl, and brine. The collected organic layer was dried over anhydrous sodium sulfate, filtered, and concentrated under reduced pressure. Silica gel, *n*-hexane/ethyl acetate 4:6; 35%; 122°C; IR (cm^-1^) 3498 (CONH amide ), 1673 (CO aldehyde), 1641 (CO ketone); ^1^H NMR (DMSO-*d*_6_, 400 MHz) *δ* (ppm) 3.96 (s, 3H, CH_3_), 5.60 (s, 2H, CH_2_), 7.16-7.20 (m, 2H, benzene H), 7.28-7.33 (m, 3H, benzene H), 7.64 (bs, 1H, CH pyrrole), 7.89 (d, 2H, *J_o_* = 8 Hz, benzene H), 7.98 (d, 2H, *J_o_* = 8 Hz, benzene H), 8.05 (d, 1H, *J_o_* = 8 Hz, benzene H), 8.19 (bs, 1H, CH pyrrole), 8.27 (s, 1H, benzene H), 9.64 (s, 1H, aldehyde CH), 10.50 (s, 1H, NH). Anal. Calcd for C_27_H_20_BrFN_2_O_4_: C, 60.57; H, 3.77; Br, 14.93; F, 3.55; N, 5.23%. Found C, 60.54; H, 3.76; Br, 14.91; F, 3.55; N, 5.22%.

**4-benzoyl-1-(4-chlorobenzyl)-1*H*-pyrrole-2-carbaldehyde (14h).** Compound **14h** was prepared by means GP B using 4-benzoyl-1*H*-pyrrole-2-carbaldehyde^3^ and 4-chlorobenzyl chloride as alkylating agent. 5h, Silica gel, *n*-hexane/ethyl acetate 3:7; 70%; 152°C; IR (cm^-1^) 1650 (CO aldehyde), 1635 (CO ketone); ^1^H NMR (DMSO-*d*_6_, 400 MHz) *δ* (ppm) 5.62 (s, 2H, CH_2_), 7.20 (d, 2H, *J_o_* = 8 Hz, benzene H), 7.35-7.38 (m, 2H, CH pyrrole and benzene H), 7.47 (m, 1H, CH pyrrole), 7.55 (t, 2H, *J_o_* = 8 Hz, benzene H), 7.65 (m, 2H, benzene H), 7.88 (m, 2H, benzene H), 9.67 (s, 1H, aldehyde CH). Anal. Calcd for C_19_H_14_ClNO_2_: C, 70.48; H, 4.36; Cl, 10.95; N, 4.33%. Found C, 70.44; H, 4.35; Cl, 10.93; N, 4.32%.

**4-benzoyl-1-(4-methoxybenzyl)-1*H*-pyrrole-2-carbaldehyde (14i).** Compound **14i** was prepared by means GP B using 4-benzoyl-1*H*-pyrrole-2-carbaldehyde^3^ and 4-methoxybenzyl chloride as alkylating agent. 5 h, Silica gel, *n*-hexane/ethyl acetate 3:7; 82%; 148°C; IR (cm^-1^) 1668 (CO aldehyde), 1635 (CO ketone); ^1^H NMR (DMSO-*d*_6_, 400 MHz) *δ* (ppm) 3.53 (s, 3H, OCH_3_), 5.58 (s, 2H, CH_2_), 7.18 (d, 2H, *J_o_* = 8 Hz benzene H), 7.25 (d, 2H, *J_o_* = 8 Hz benzene H), 7.32-7.45 (m, 3H, CH pyrrole and benzene H), 7.62-7.66 (m, 3H, benzene H), 8.17 (s, 1H, CH pyrrole), 9.62 (s, 1H, aldehyde CH). Anal. Calcd for C_20_H_17_NO_3_: C, 75.22; H, 5.37; N, 4.39%. Found C, 75.18; H, 5.36; N, 4.38%.

**4-benzoyl-1-(naphthalen-1-ylmethyl)-1*H*-pyrrole-2-carbaldehyde (14j).** Compound **14j** was prepared by means GP B using 4-benzoyl-1*H*-pyrrole-2-carbaldehyde^3^ and 1-(chloromethyl)naphthalene. 8 h; 60%; IR (cm^-1^) 1672 (CO aldehyde), 1634 (CO ketone); ^1^H NMR (DMSO-*d*_6_, 400 MHz) *δ* (ppm) 5.84 (s, 2H, CH_2_), 7.43-7.46 (m, 1H, benzene H), 7.53-7.56 (m, 2H, benzene H), 7.60-7.69 (m, 3H, benzene H), 7.73-7.89 (m, 2H, benzene H and CH pyrrole), 7.90-7.95 (m, 5H, benzene H), 8.27 (s, 1H, CH pyrrole), 9.70 (s, 1H, aldehyde CH). Anal. Calcd for C_23_H_17_NO_2_: C, 81.40; H, 5.05; N, 4.13%. Found C, 81.36; H, 5.04; N, 4.13%.

**Synthesis of 4-benzoyl-1-((4-fluorophenyl)sulfonyl)-1*H*-pyrrole-2-carbaldehyde (14m).** A solution of 4-benzoyl-1*H*-pyrrole-2-carbaldehyde^3^ (1 g, 4.69 mmol) in 15 mL of dry THF was cooled at 0°C under inert atmosphere and NaH (60 % dispersion in mineral oil) (0.36 g, 7.03 mmol) was added slowly. After 10 min, 4-fluorobenzene-1-sulfonyl chloride (1.22 g, 6.1 mmol) was added dropwise at 0°C. The reaction mixture was slowly warmed up to room temperature and stirred for 12h. Upon reaction completion, checked by TLC, the solution was reduced under pressure, and water and dichloromethane were added (200 mL). The aqueous layer was further extracted with dichloromethane. The organic fractions were washed with brine, dried over anhydrous sodium sulfate, and concentrated under reduced pressure. The solid was washed with *n*-hexane (2 x 10 mL) and light petroleum ether (2 x 10 mL)**.** Silica gel, *n*-hexane/ethyl acetate 3:7; 167°C; IR (cm^-1^) 1669 (CO aldehyde), 1633 (CO ketone); ^1^H NMR (DMSO-*d*_6_, 400 MHz) *δ* (ppm) 7.19 (s, 1H, CH pyrrole), 7.47-7.82 (m, 10H, CH pyrrole, and benzene H), 9.45 (s, 1H, CH aldehyde). Anal. Calcd for C_18_H_12_FNO_4_S: C, 60.50; H, 3.38; F, 5.32; N, 3.92; S, 8.97%. Found C, 60.45; H, 3.38; F, 5.31; N, 3.91; S, 8.96%.

**(*E*)-4-(4-(2,3-dihydrobenzo[*b*][1,4]dioxine-6-carbonyl)-1-(4-fluorobenzyl)-1*H*-pyrrol-2-yl)but-3-en-2-one (15c).** Compound **15c** was prepared by means GP C using **14c**. Silica gel, chloroform/ethyl acetate 1:1; 65%; 125 °C; IR (cm^-1^) 1670 (CO ketone), 1635 (CO enone); ^1^H NMR (DMSO-*d*_6_, 400 MHz) *δ* (ppm) 2.12 (s, 3H, butenoate C1-H), 4.23 (s, 4H, CH_2_, 1,4-dioxane), 5.40 (s, 2H, CH_2_), 6.52 (d, 1H, *J_t_* = 16 Hz, butenoate C3-H), 6.90 (d, 2H, *J_o_* = 8 Hz, benzene H), 7.09-7.17 (m, 5H, CH pyrrole and benzene H), 7.23 (s, 1H, benzene H), 7.28-7.37 (m, 1H, benzene H), 7.33 (d, 1H, *J_t_* = 16 Hz, butenoate C4-H), 7.82 (s, 1H, CH pyrrole). Anal. Calcd for C_24_H_20_FNO_4_: C, 71.10; H, 4.97; F, 4.69; N, 3.45%. Found C, 71.06; H, 4.96; F, 4.69; N, 3.45%.

**(*E*)-4-(1-(4-fluorobenzyl)-4-(4-(naphthalen-2-ylmethoxy)benzoyl)-1*H*-pyrrol-2-yl)but-3-en-2-one (15d).** Compound **15d** was prepared by means GP C using **14d**. Silica gel, chloroform/ethyl acetate 1:1; 72%; 149 °C; IR (cm^-1^) 1672 (CO ketone), 1655 (CO enone); ^1^H NMR (DMSO-*d*_6_, 400 MHz) *δ* (ppm) 2.21 (s, 3H, butenoate C1-H), 5.40 (s, 2H, CH_2_), 5.48 (s, 2H, CH_2_), 6.64 (d, 1H, *J_t_* = 16.00 Hz, butenoate C3-H), 7.17-7.28 (m, 6H, benzene H, CH pyrrole and naphthalene H), 7.44 (d, 1H, *J_t_* = 16.00 Hz, butenoate C4-H), 7.52-7.63 (m, 3H, benzene H and CH pyrrole), 7.83-8.02 (m, 7H, benzene H and naphthalene H). Anal. Calcd for C_33_H_26_FNO_3_: C, 78.71; H, 5.20; F, 3.77; N, 2.78%. Found C, 78.65; H, 5.18; F, 3.76; N, 2.78%.

**(*E*)-4-(1-(4-fluorobenzyl)-4-(4-phenoxybenzoyl)-1*H*-pyrrol-2-yl)but-3-en-2-one (15e).** Compound **15e** was prepared by means GP C using **14e**. Silica gel, *n*-hexane/ethyl acetate; 72%; 152 °C; IR (cm^-1^) 1586 (CO ketone) 1698 (enone); ^1^H NMR (DMSO-*d*_6_, 400 MHz) *δ* (ppm) 2.11 (s, 3H, butenoate C1-H), 5.20 (s, 2H, CH_2_), 6.52 (d, 1H, *J_t_* = 16 Hz, butenoate C3-H), 6.83 (d, 2H, *J_O_* = 8 Hz, benzene H), 6.88-7.20 (m, 7H, CH pyrrole and benzene H), 7.32-7.39 (m, 4H, butenoate C4-H and benzene H), 7.77 (d, 2H, *J_O_* = 8 Hz, benzene H), 7.84 (s, 1H, CH pyrrole). Anal. Calcd for C_28_H_22_FNO_3_: C, 76.52; H, 5.05; F, 4.32; N, 3.19%. Found C, 76.48; H, 5.04; F, 4.31; N, 3.19%.

**(*E*)-4-(1-(4-fluorobenzyl)-4-(4-nitrobenzoyl)-1*H*-pyrrol-2-yl)but-3-en-2-one (15f).** Compound **15f** was prepared by means GP C using **15f**. Silica gel, *n*-hexane/ethyl acetate 6:4; 65%; 144 °C; IR (cm^-1^) 1650 (CO ketone), 1630 (CO enone); ^1^H NMR (DMSO-*d*_6_, 400 MHz) *δ* (ppm) 2.22 (s, 3H, butenoate C1-H), 5.29 (s, 2H, CH_2_), 6.65 (d, 1H, *J_t_* = 16 Hz, butenoate C3-H), 7.18-7.26 (m, 4H, benzene H), 7.32 (s, 1H, CH pyrrole), 7.43 (d, 1H, *J_t_* = 16 Hz, butenoate C4-H), 7.96 (m, 3H, CH pyrrole and benzene H), 8.35 (d, 2H, *J_o_* = 8 Hz, benzene H). Anal. Calcd for C_22_H_17_FN_2_O_4_: C, 67.34; H, 4.37; F, 4.84; N, 7.14%. Found C, 67.30; H, 4.36; F, 4.83; N, 7.13%.

**(*E*)-3-bromo-*N*-(4-(1-(4-fluorobenzyl)-5-(3-oxobut-1-en-1-yl)-1*H*-pyrrole-3 carbonyl)phenyl)-4-methoxybenzamide (15g).** Compound **15g** was prepared by means GP C using **14g**. Silica gel, n-hexane/ethyl acetate 4:6; 74%; 113 °C; IR (cm^-1^) 3220 (CONH amide), 1630 (CO ketone); ^1^H NMR (DMSO-*d*_6_, 400 MHz) *δ* (ppm) 2.22 (s, 3H, butenoate C1-H), 3.96 (s, 3H, CH_3_), 5.50 (s, 2H, CH_2_), 6.64 (d, 1H, *J_t_* = 16 Hz, butenoate C3-H), 7.19-7.31 (m, 6H, benzene H and CH pyrrole), 7.44 (d, 1H, *J_t_* = 16 Hz, butenoate C4-H), 7.86-8.06 (m, 6H, benzene H), 8.26 (s, 1H, CH pyrrole), 10.48 (s, 1H, NH). Anal. Calcd for C_30_H_24_BrFN_2_O_4_: C, 62.62; H, 4.20; Br, 13.89; F, 3.30; N, 4.87%. Found C, 62.58; H, 4.19; Br, 13.87; F, 3.29; N, 4.87%.

**(*E*)-4-(4-benzoyl-1-(4-chlorobenzyl)-1*H*-pyrrol-2-yl)but-3-en-2-one (15h).** Compound **15h** was prepared by means GP C using 14h. Silica gel, chloroform/ethyl acetate 1:1; 74%; 132 °C; IR (cm^-1^) 1703 (CO ketone), 1655 (CO enone); ^1^H NMR (DMSO-*d*_6_, 400 MHz) *δ* (ppm) 2.26 (s, 3H, butenoate C1-H), 4.71 (s, 2H, CH_2_), 6.69 (d, 1H, *J_t_* = 16 Hz, butenoate C3-H), 7.25 (d, 2H, *J_o_* = 8 Hz, benzene H), 7.27 (s, 1H, CH pyrrole), 7.36-7.51 (m, 2H, butenoate C4-H and benzene H), 7.58-7.60 (m, 2H, benzene H), 7.62-7.67 (m, 2H, benzene H), 7.87 (d, 2H, *J_o_* = 8 Hz, benzene H), 7.98 (s, 1H, CH pyrrole). Anal. Calcd for C_22_H_18_ClNO_2_: C, 72.63; H, 4.99; Cl, 9.74; N, 3.85%. Found C, 72.59; H, 4.98; Cl, 9.72; N, 3.85%.

**(*E*)-4-(4-benzoyl-1-(4-methoxybenzyl)-1*H*-pyrrol-2-yl)but-3-en-2-one (15i).** Compound **15i** was prepared by means GP C using **14i.** Silica gel, chloroform/ethyl acetate 1:1; 70%; 138 °C; IR (cm^-1^) 1667 (CO ketone), 1632 (CO enone), 1250 (C-O-C), 1040 (C-O-C); ^1^H NMR (DMSO-*d*_6_, 400 MHz) *δ* (ppm) 2.22 (s, 3H, butenoate C1-H), 3.75 (s, 3H, OCH_3_), 5.39 (s, 2H, CH_2_), 6.89-6.91 (m, 3H, butenoate C3-H and benzene H), 7.12 (d, 2H, *J_o_* = 8 Hz, benzene H), 7.38 (s, 1H, CH pyrrole), 7.55-7.57 (m, 2H, benzene H), 7.60-7.63 (m, 2H, butenoate C4-H and benzene H), 7.79 (d, 2H, *J_o_* = 8 Hz benzene H), 7.93 (s, 1H, CH pyrrole). Anal. Calcd for C_23_H_21_NO_3_: C, 76.86; H, 5.89; N, 3.90%. Found C, 76.82; H, 5.87; N, 3.89%.

**(*E*)-4-(4-benzoyl-1-(naphthalen-1-ylmethyl)-1*H*-pyrrol-2-yl)but-3-en-2-one (15j).** Compound **15j** was prepared by means GP C using **14j**. Silica gel, n-hexane/ethyl acetate 1:1; 74%; 195 °C; IR (cm^-1^) 1648 (CO ketone), 1630 (CO enone); ^1^H NMR (DMSO-*d*_6_, 400 MHz) *δ* (ppm) 2.19 (s, 3H, butenoate C1-H), 6.10 (s, 2H, CH_2_), 6.75 (d, 1H, *J_t_* = 16 Hz, butenoate C3-H), 6.85 (d, 2H, *J_o_* = 8 Hz, benzene H), 7.54-7.58 (m, 2H, CH pyrrole and benzene H), 7.62-7.67 (m, 2H, benzene H), 7.68-7.78 (m, 3H, butanoate C4-H and benzene H), 7.86-7.89 (m, 3H, CH pyrrole and benzene H), 7.91 (d, 1H, *J_o_* = 8 Hz, benzene H), 8.07 (d, 1H, *J_o_* = 8 Hz, benzene H), 8.23 (d, 1H, *J_o_* = 8 Hz, benzene H). Anal. Calcd for C_26_H_21_NO_2_: C, 82.30; H, 5.58; N, 3.69%. Found C, 82.26; H, 5.57; N, 3.68%.

**(*E*)-4-(4-(3,4,5-trimethoxybenzoyl)-1*H*-pyrrol-2-yl)but-3-en-2-one (15k).** Compound **15k** was prepared by means GP C using **13k**. Silica gel, *n*-hexane/ethyl acetate 2:8; 74%; 144 °C; IR (cm^-1^) 1662 (CO ketone), 1635 (CO enone), 1221 (C-O-C), 1059 (C-O-C); ^1^H NMR (DMSO-*d*_6_, 400 MHz) *δ* (ppm) 2.16 (s, 3H, butenoate C1-H), 3.65 (s, 3H, OCH_3_), 3.74 (s, 6H, OCH_3_), 6.51-6.55 (d, 1H, *J_t_* = 16.0 Hz, butenoate C3-H), 6.97 (s, 3H, benzene H and CH pyrrole), 7.36-7.40 (d, 1H, *J_t_* = 16.0 Hz, butenoate C4-H), 7.65 (s, 1H, CH pyrrole), 12.16 (s, 1H, NH). Anal. Calcd for C_18_H_19_NO_5_: C, 65.64; H, 5.82; N, 4.25%. Found C, 65.60; H, 5.81; N, 4.25%.

**(*E*)-4-(4-benzoyl-1-((4-fluorophenyl)sulfonyl)-1*H*-pyrrol-2-yl)but-3-en-2-one (15m).** Compound **15m** was prepared by means GP C using **14m**. Silica gel, chloroform/ethyl acetate 1:1; 68%; 116 °C; IR (cm^-1^) 1660 (CO ketone), 1633 (CO enone); ^1^H NMR (DMSO-*d*_6_, 400 MHz) *δ* (ppm) 2.30 (s, 3H, butenoate C1-H), 6.88 (d, 1H, *J_t_* = 16 Hz, butenoate C3-H), 7.52-7.62 (m, 4H, CH pyrrole, butenoate C4-H and benzene H), 7.70-7.75 (m, 2H, benzene H), 7.88 (d, 2H, *J_o_* = 8.0 Hz, benzene H), 8.15-8.21 (m, 4H, CH pyrrole and benzene H). Anal. Calcd for C_21_H_16_FNO_4_S: C, 63.47; H, 4.06; F, 4.78; N, 3.52; S, 8.07%. Found C, 63.42; H, 4.05; F, 4.77; N, 3.52; S, 8.06%.

**Synthesis of 4-(4-aminobenzoyl)-1-(4-fluorobenzyl)-1*H*-pyrrole-2-carbaldehyde (16).** NHEt_2_ (0.20 g, 2.84 mmol), Pd/C 10% (10% w/w), and ammonium formate (0.88 g, 14 mmol) were added to a well-stirred solution of **14f** (1 g, 2.84 mmol) in ethyl acetate, and it was refluxed for 1 h under argon atmosphere. The reaction was quenched with water, cooled to room temperature, filtered on Celite, and washed with ethyl acetate. The organic layer was evaporated under vacuum, and the raw material was extracted with ethyl acetate and water. The organic phase was separated, washed with brine, dried over anhydrous sodium sulfate, filtered, and evaporated under vacuum to afford the pure aniline in a quantitative yield The compound was used in the subsequent step without further purification and characterization.

**Synthesis of (*E*)-4-(4-(3,4,5-trimethoxyphenyl)-1*H*-pyrrol-3-yl)but-3-en-2-one (17).** To a well stirred suspension of NaH 60% (0.9 g, 22 mmol) in 86 mL of anhydrous diethyl ether under argon atmosphere, a solution of (3*E*,5*E*)-6-(3,4,5-trimethoxyphenyl)hexa-3,5-dien-2-one (2.7 g, 10 mmol)^5^ and TosMIC (2.21 g, 11 mmol) in diethyl ether/DMSO 2:1 (170 mL) was added dropwise at room temperature. After the addition, the reaction mixture was stirred at room temperature for 30 minutes. the mixture was diluted with water and extracted with ethyl acetate. The combined organic phases were washed with brine, dried over anhydrous sodium sulfate, and evaporated at reduced pressure. The crude product was purified by column chromatography (silica gel/ethyl acetate) to furnish 1.88 g of pure derivative **17** (62%). IR (cm^-1^) 1647 (CO enone), 1225 (C-O-C), 1043 (C-O-C); ^1^H NMR (DMSO-*d*_6_, 400 MHz) *δ* (ppm) 2.36 (s, 3H, CH_3_), 3.63 (s, 3H, OCH_3_), 3.83 (s, 6H, OCH_3_), 6.71 (s, 2H, benzene H), 6.88 (d, 1H, *J_t_* = 16 Hz, C3-H), 7.24 (s, 1H, CH pyrrole), 7.57 (d, 1H, *J_t_* = 16 Hz, C4-H), 7.68 (s, 1H, CH pyrrole), 11.55 (s, 1H, NH). Anal. Calcd for C_17_H_19_NO_4_: C, 67.76; H, 6.36; N, 4.65%. Found C, 67.73; H, 6.34; N, 4.65%.

**Table S1.** Pol λ and pol β residual activity after treatment 100 μM of diketo hexenoic derivatives **5a**-**g,j-m**, **6a**-**g,j-m**, **7a**-**d**, **8a**-**d** and diketo butanoic derivatives **9a**,**b**, **10a**,**b**, **11a**,**b** and **12a**,**b**.

| **Cpd** | **pol λ (Pol) Residual activity (%)*^a^*** | | **pol λ (TdT) Residual activity (%)*^b^*** | **pol** β **Residual activity (%)*^c^*** | |
| --- | --- | --- | --- | --- | --- |
|  | **Mg^2+^** | **Mn^2+^** | **Mn^2+^** | **Mg^2+^** | **Mn^2+^** |
| **5a** | 100% | 100% | nd*^d^* | 53% | 100% |
| **5b** | 95% | 100% | 45% | 88% | 95% |
| **5c** | 100% | 100% | 100% | nd | nd |
| **5d** | 27.1% | 70.3% | nd | 10% | 42.7% |
| **5e** | nd | nd | nd | nd | nd |
| **5f** | 60.5% | 75.3% | nd | 5.1% | 33.3% |
| **5g** | 71.3% | 100% | nd | 10% | 100% |
| **5j** | 32.6% | 100% | nd | 23.7% | 100% |
| **5k** | 60.7% | 63.9% | 50.4% | 68% | 100% |
| **5l** | 75.2% | 100% | nd | 52% | 100% |
| **5m** | 20.4% | 64.7% | 100% | 29% | 84.9% |
| **6a** | 90.0% | 77% | 29.6% | 35% | 50.2% |
| **6b** | 84.0% | 98.0% | 50.0% | 81.9% | 92% |
| **6c** | 100% | 100% | 100% | nd | nd |
| **6d** | 33.1% | 100% | nd | 4.44% | 62% |
| **6e** | nd | nd | nd | nd | nd |
| **6f** | 17.9% | 52.1% | nd | 1.88% | 22% |
| **6g** | 89.0% | 100% | nd | 4.14% | 100% |
| **6j** | 15.1% | 100% | nd | 27.1% | 100% |
| **6k** | 37.7% | 20.6% | 42.5% | 73% | 100% |
| **6l** | 40.5% | 100% | nd | 40.1% | 100% |
| **6m** | 11.4% | 21.3% | 50.0% | 45.5% | 62.7% |
| **7a** | 96.0% | 100% | 77% | 82.4% | 95% |
| **7b** | 100% | 100% | 100% | 100% | 69.7% |
| **7c** | 42.3% | 100% | nd | 57% | 100% |
| **7d** | 44.6% | 100% | nd | 67.5% | 100% |
| **8a** | 93.0% | 100% | 59% | 84% | 95% |
| **8b** | 54.6% | 90.7% | 30% | 70.7% | 100% |
| **8c** | 15.6% | 50.7% | nd | 70% | 100% |
| **8d** | 43.2% | 100% | nd | 54.6% | 100% |
| **9a** | 69.0% | 82.3% | 100% | 84% | 100% |
| **9b** | 100% | 100% | 30.1% | 100% | 100% |
| **10a** | 100% | 100% | 57.7% | 86% | 67% |
| **10b** | 93% | 100% | 81.0% | 39.7% | 68.3% |
| **11a** | 100% | 100% | 100% | 94% | 98% |
| **11b** | 70.0% | 94.0% | nd | 60.5% | 88% |
| **12a** | 100% | 100% | 30.1% | 100% | 100% |
| **12b** | 74.0% | 79.0% | 100% | 51.7% | 82.5% |

*^a^*Percentage of pol **λ** polymerase residual activity (%) in presence of Mg^2^**^+^** or Mn^2+^. *^b^*Percentage of pol **λ** TdT residual activity (%) in presence of Mn^2+^. *^c^*Percentage of pol β residual activity (%) in presence of Mn^2+^. *^d^*nd: not determined.

**Figure S1**. MolProbity^6^ performed a Ramachandran plot analysis, revealing that 98.6% (354/359) of all residues were in favored (98%) regions. 100.0% (359/359) of all residues were in allowed (>99.8%) regions.

There were no outliers. As a result, the model was validated as being of high quality and was used for computational simulation of ligand-receptor interactions.


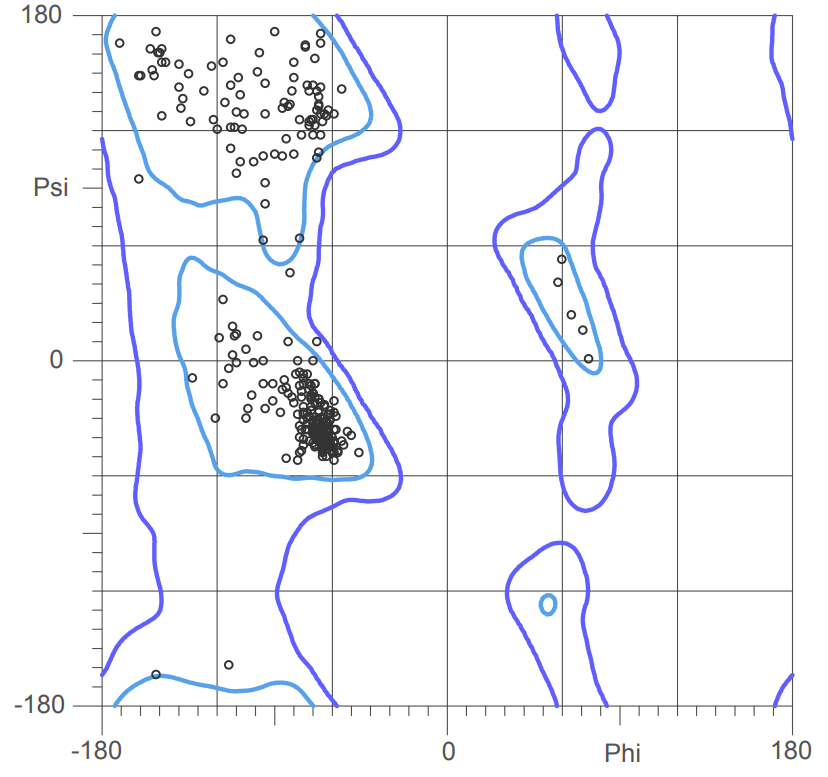


**Figure S2**. Binding mode prediction of reference compound **4** in complexes with Mg^2+^ and Mn^2+^ ion, reported in green and yellow, respectively. Sodium are reported as a purple sphere; nucleic acid is reported as orange stick.

**
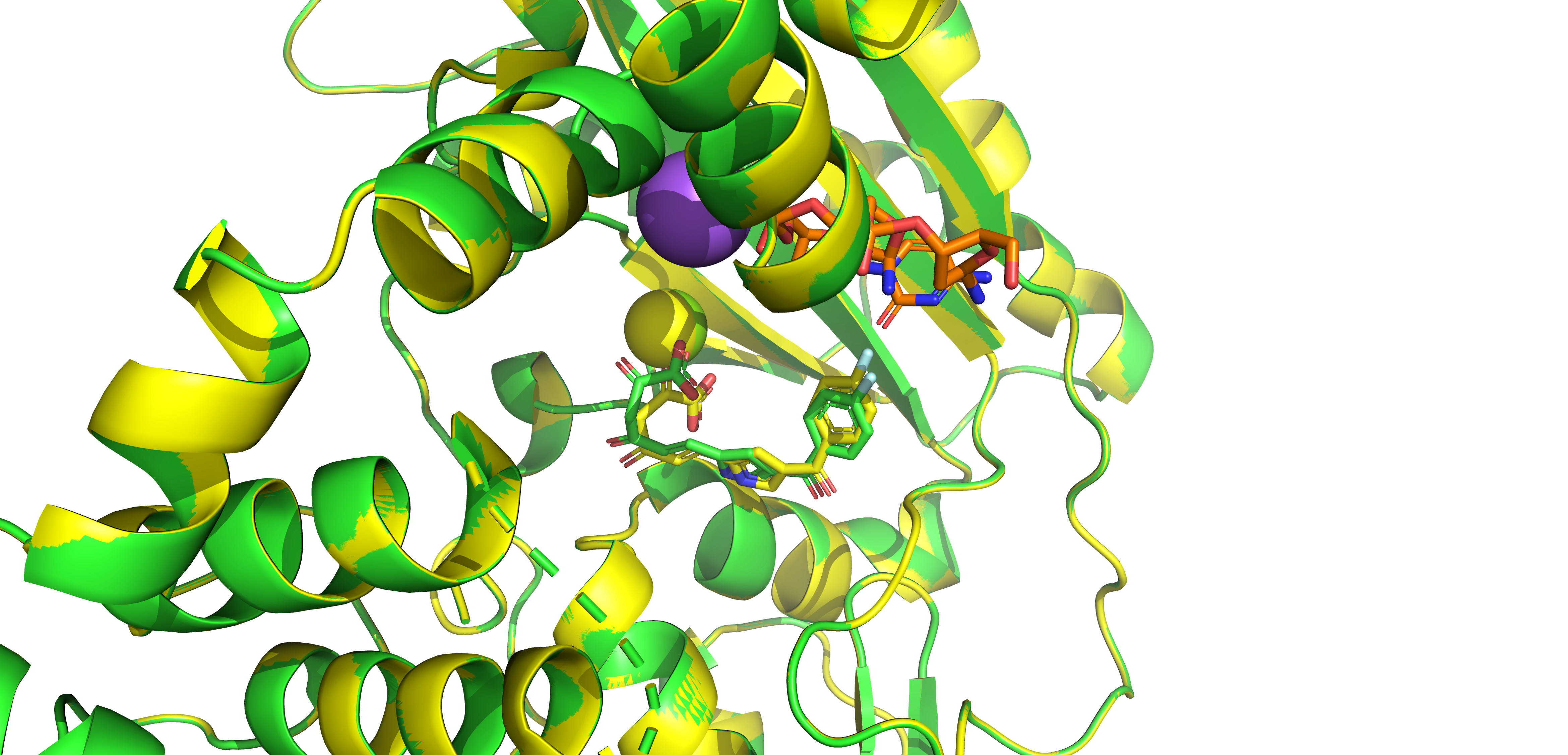
**

REFERENCES

1. Costi, R.; Metifiot, M.; Esposito, F.; Cuzzucoli Crucitti, G.; Pescatori, L.; Messore, A.; Scipione, L.; Tortorella, S.; Zinzula, L.; Novellino, E.; Pommier, Y.; Tramontano, E.; Marchand, C.; Di Santo, R. 6-(1-Benzyl-1H-pyrrol-2-yl)-2,4-dioxo-5-hexenoic acids as dual inhibitors of recombinant HIV-1 integrase and ribonuclease H, synthesized by a parallel synthesis approach. *J. Med. Chem.*, **2013**, *56*, 8588−8598. doi.org/10.1021/jm401040b.
2. Cuzzucoli Crucitti, G.; Metifiot, M.; Pescatori, L.; Messore, A.; Madia, V. N.; Pupo, G.; Saccoliti, F.; Scipione, L.; Tortorella, S.; Esposito, F.; Corona, A.; Cadeddu, M.; Marchand, C.; Pommier, Y.; Tramontano, E.; Costi, R.; Di Santo, R. Structure-activity relationship of pyrrolyl diketo acid derivative as dual inhibitors of HIV-1 integrase and reverse transcriptase ribonuclease H domain. *J. Med. Chem.*, **2015**, *58(4)*, 1915-1928. doi.org/10.1021/jm501799k.
3. Ialongo, D.; Messore, A.; Madia, V.N.; Tudino, V.; Nocentini, A.; Gratteri, P.; Giovannuzzi, S.; Supuran, C.T.; Nicolai, A.; Scarpa, S.; Taurone, S.; Camarda, M.; Artico, M.; Papa, V.; Saccoliti, F.; Scipione, L.; Di Santo, R.; Costi, R. Pyrrolyl and Indolyl α-γ-Diketo Acid Derivatives Acting as Selective Inhibitors of Human Carbonic Anhydrases IX and XII. *Pharmaceuticals*, **2023**, *16(2)*, 188. doi: 10.3390/ph16020188.
4. Wang, J.; Ge, Y.Q.; Jia, J.; Yang, H.; Zhao, G.L.; Zhan, F.X. A Facile Approach to Indolizines via Tandem Reaction. *Heterocycles*, **2009**, *78*, 725–736. doi.org/10.3987/COM-08-11570.
5. Ishiwata, H.; Kabeya, M.; Shiratsuchi, M.; Hattori, Y.; Nakao, H.; Nagoya, T.; Sato, S.; Oda, S.; Suda, M.; Shibasaki, M. Novel Diamide Compounds and Drugs Containing the Same. PCT Int. Pat. Appl. **1998**, WO1998016497.
6. Williams, C.J.; Headd, J.J.; Moriarty, N.W.; Prisant, M.G.; Videau, L.L.; Deis, L.N.; Verma, V.; Keedy, D.A.; Hintze, B. J.; Chen, V.B.; Jain, S.; Lewis, S.M.; Arendall, W.B., 3rd; Snoeyink, J.; Adams, P.D.; Lovell, S.C.; Richardson, J.S.; Richardson, D.C. MolProbity: More and better reference data for improved all-atom structure validation. Protein science: a publication of the Protein Society. **2018**, 27(1), 293–315. doi.org/10.1002/pro.3330.

Figure S3: ^1^H NMR Spectrum for compound **5c**

Figure S4: FTIR Spectrum for compound **5c**

Figure S5: ^1^H NMR Spectrum for compound **6c**

Figure S6: FTIR Spectrum for compound **6c**

Figure S7: ^1^H NMR Spectrum for compound **5d**

**
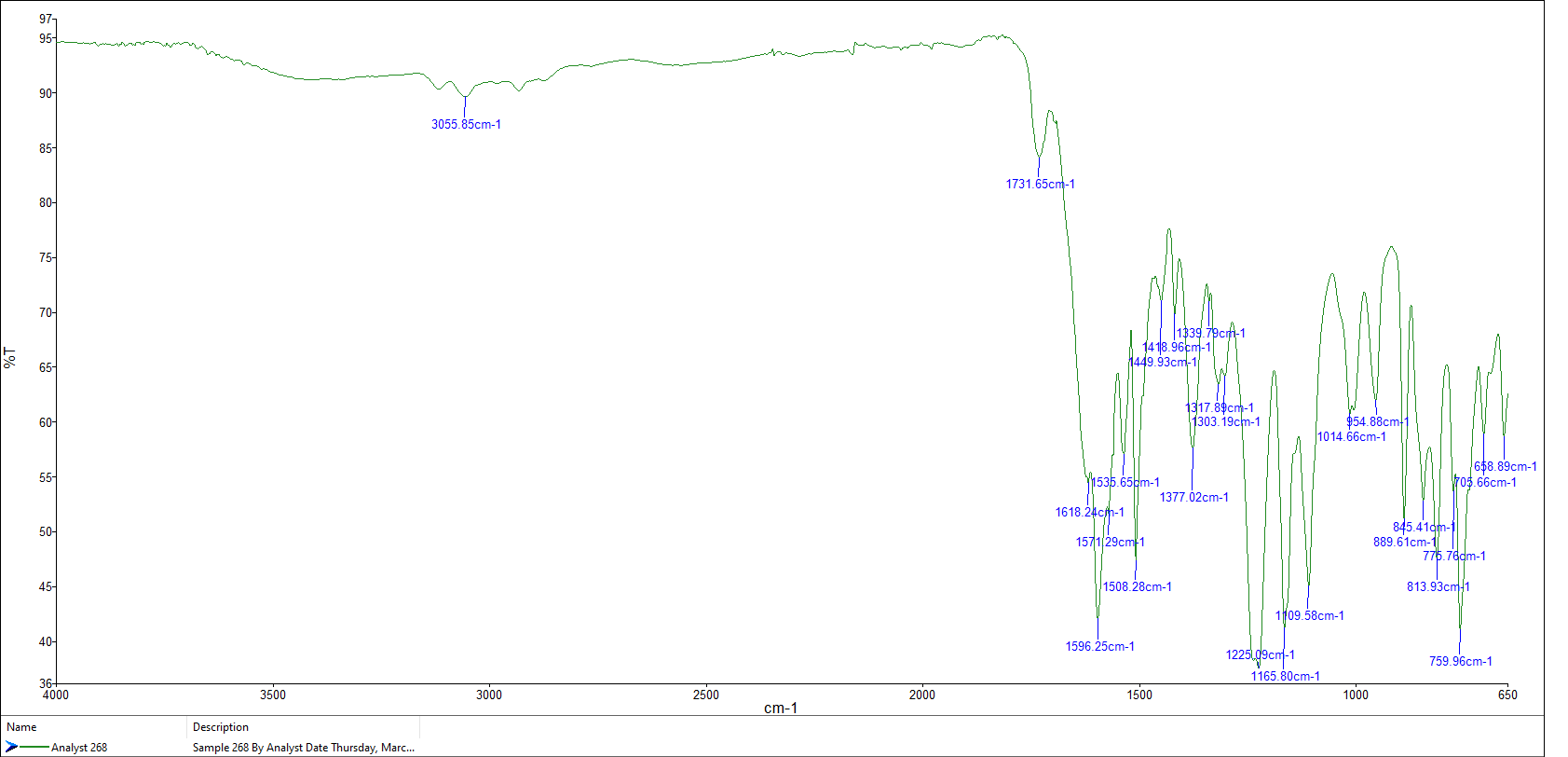
**

Figure S8: FTIR Spectrum for compound **5d**

Figure S9: ^1^H NMR Spectrum for compound **6d**

**
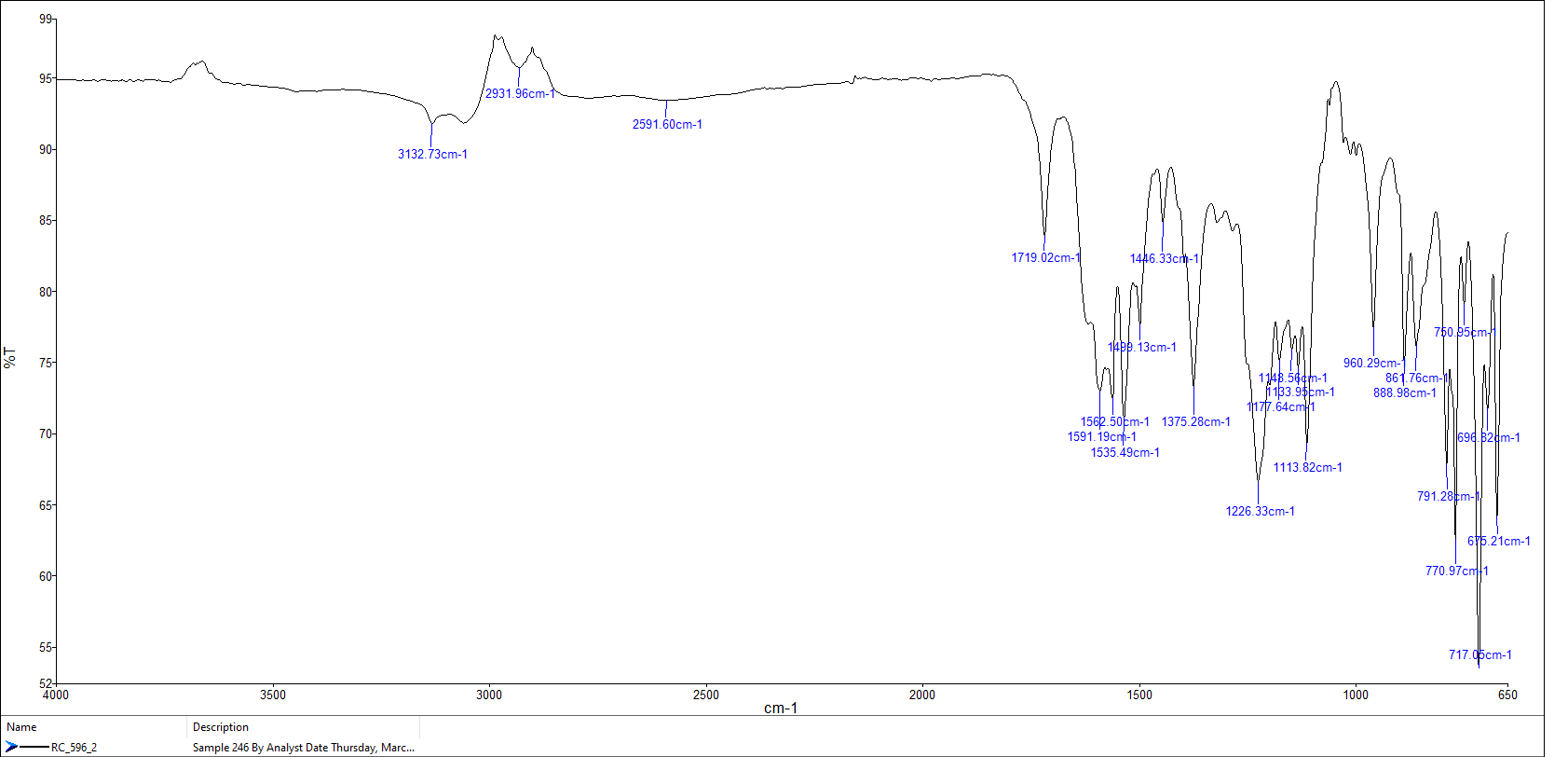
**

Figure S10: FTIR Spectrum for compound **6d**

Figure S11: ^1^H NMR Spectrum for compound **5e**

Figure S12: FTIR Spectrum for compound **5e**

Figure S13: ^1^H NMR Spectrum for compound **6e**

Figure S14: FTIR Spectrum for compound **6e**

Figure S15: ^1^H NMR Spectrum for compound **5f**

Figure S16: FTIR Spectrum for compound **5f**

Figure S17: ^1^H NMR Spectrum for compound **6f**

**
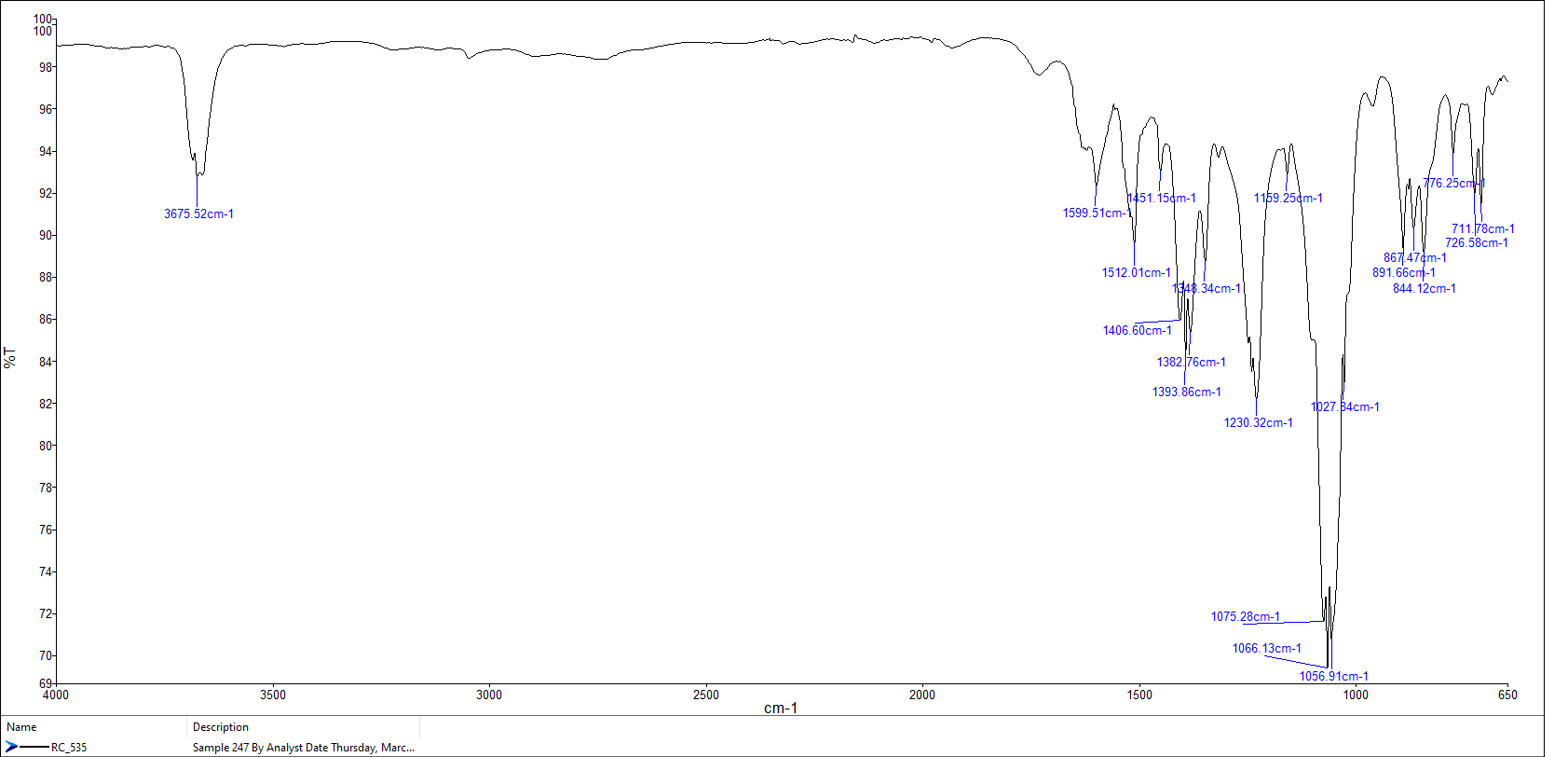
**

Figure S18: FTIR Spectrum for compound **6f**

Figure S19: ^1^H NMR Spectrum for compound **5g**

Figure S20: FTIR Spectrum for compound **5g**

Figure S21: ^1^H NMR Spectrum for compound **6g**

Figure S22: FTIR Spectrum for compound **6g**


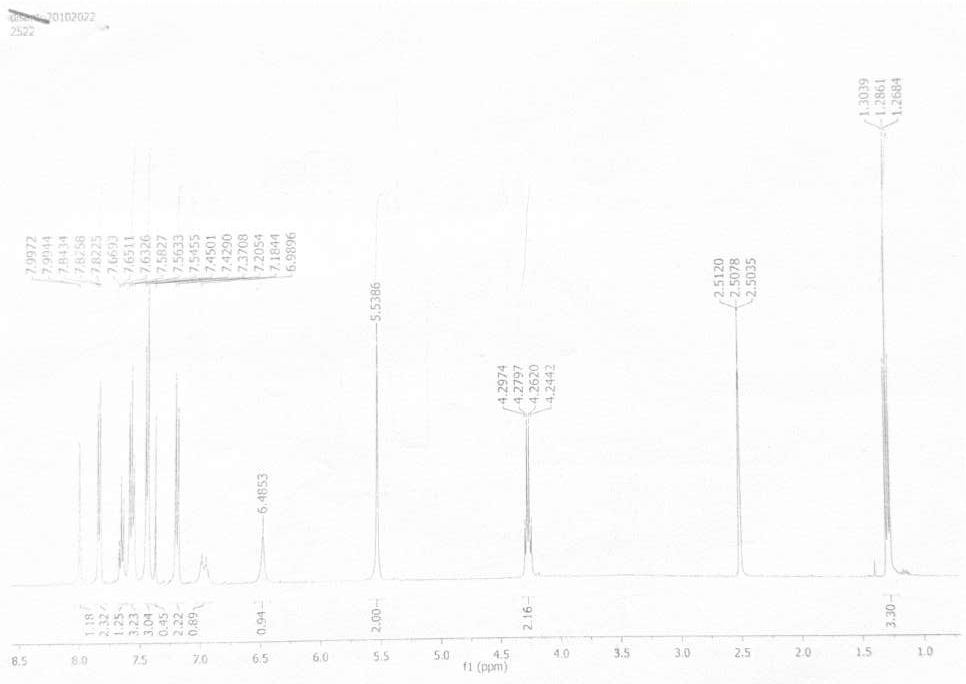


Figure S23: ^1^H NMR Spectrum for compound **5h**

Figure S24: FTIR Spectrum for compound **5h**


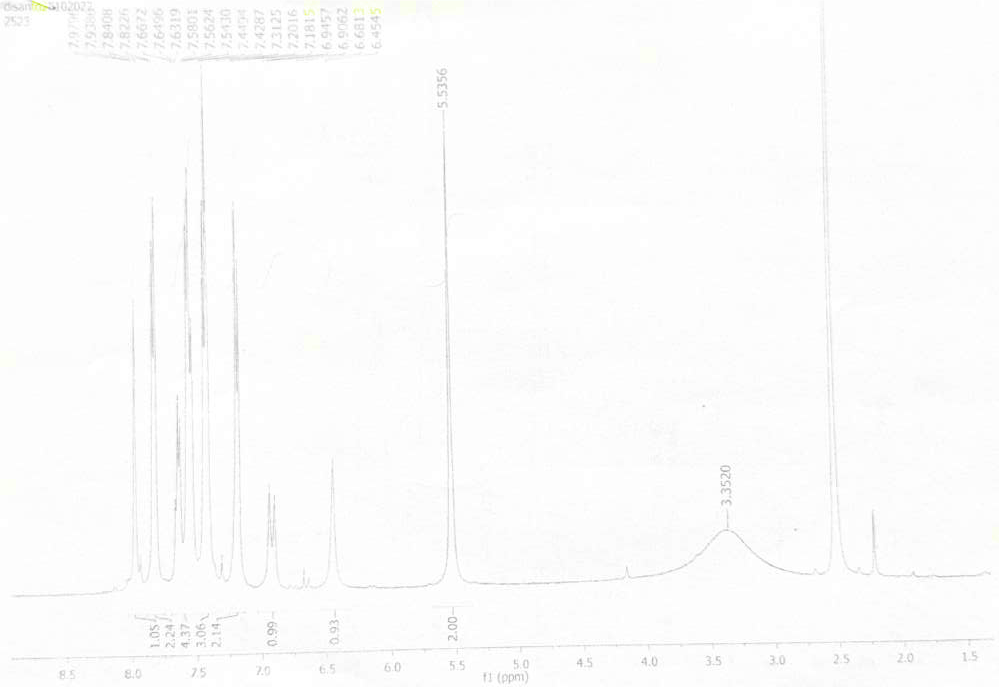


Figure S25: ^1^H NMR Spectrum for compound **6h**

Figure S26: FTIR Spectrum for compound **6h**


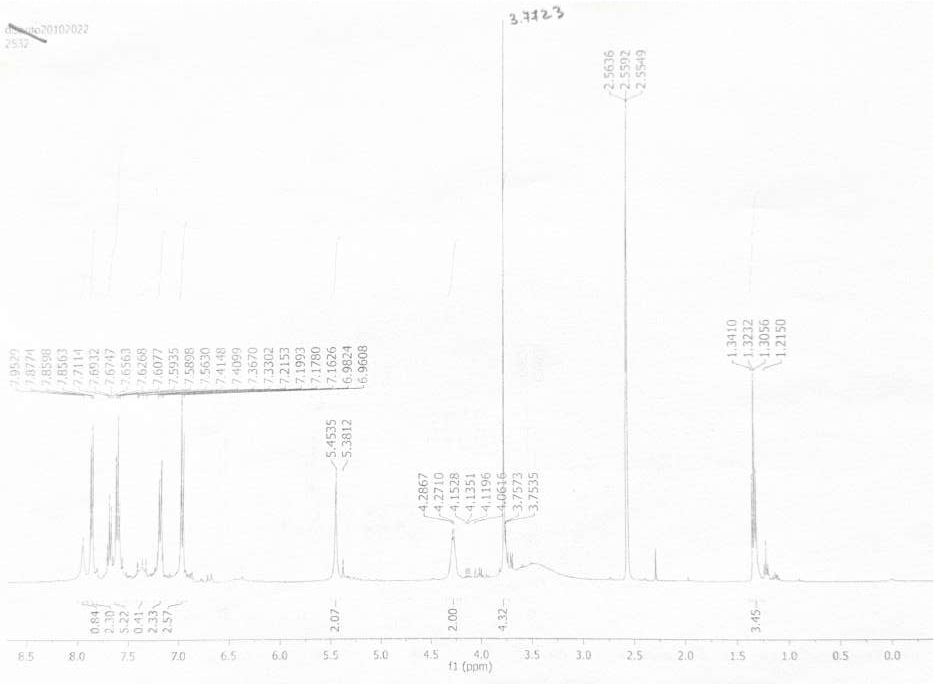


Figure S27: ^1^H NMR Spectrum for compound **5i**

Figure S28: FTIR Spectrum for compound **5i**


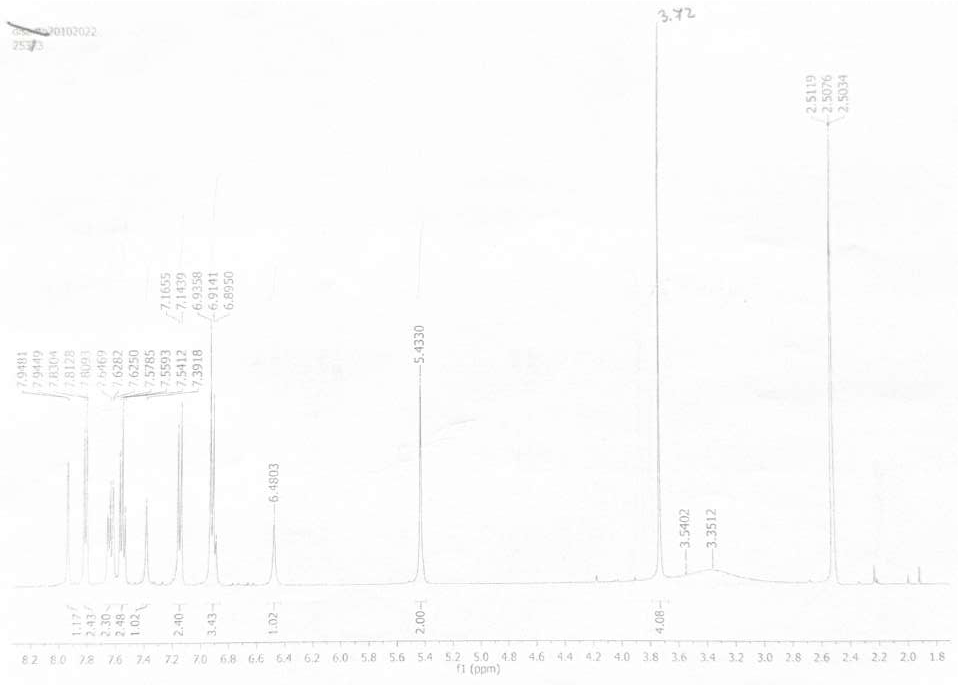


Figure S29: ^1^H NMR Spectrum for compound **6i**

Figure S30: FTIR Spectrum for compound **6i**

Figure S31: ^1^H NMR Spectrum for compound **5j**

Figure S32: FTIR Spectrum for compound **5j**

Figure S33: ^1^H NMR Spectrum for compound **6j**

Figure S34: FTIR Spectrum for compound **6j**

Figure S35: ^1^H NMR Spectrum for compound **5k**

**
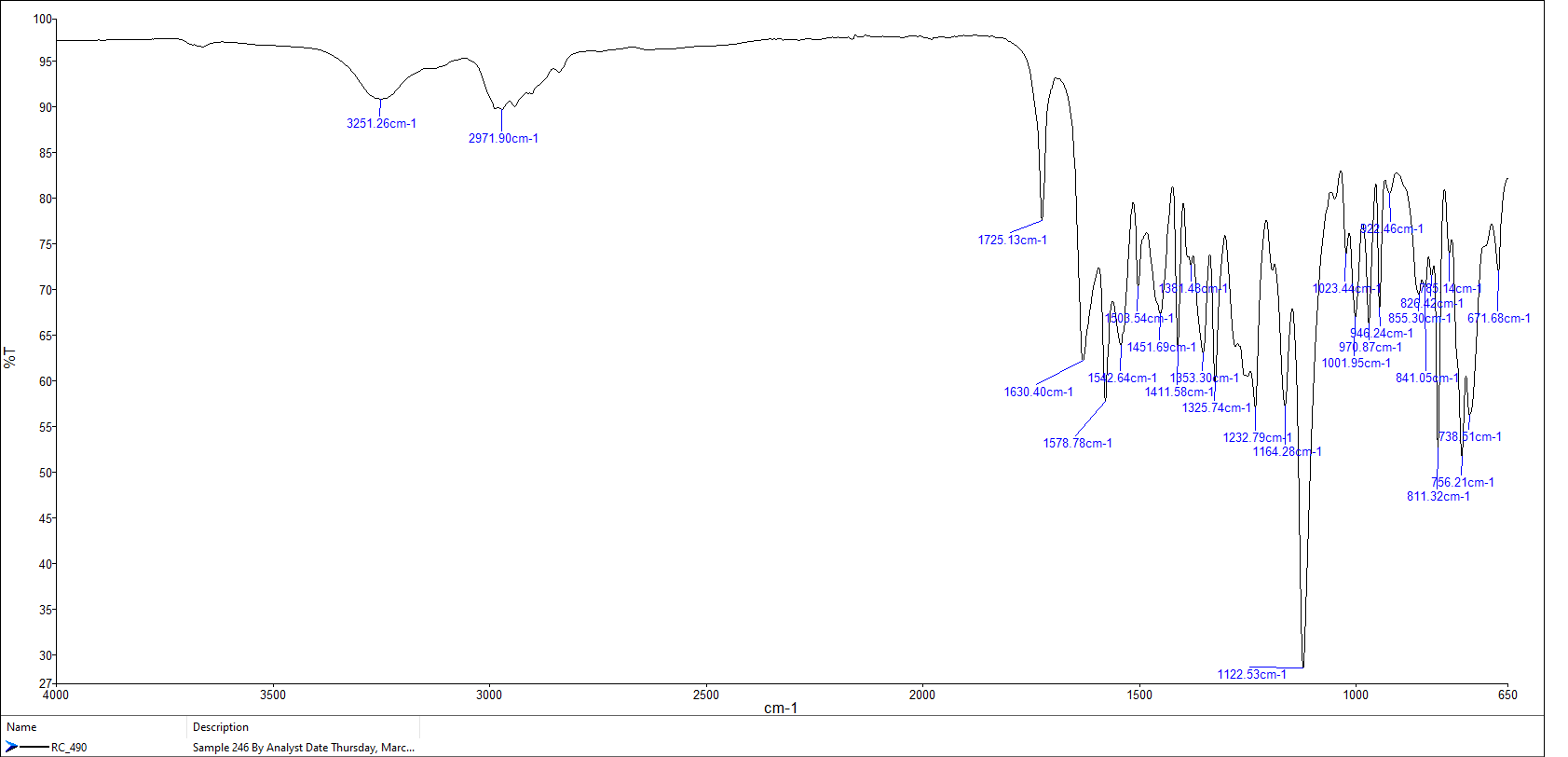
**

Figure S36: FTIR Spectrum for compound **5k**

Figure S37: ^1^H NMR Spectrum for compound **6k**

**
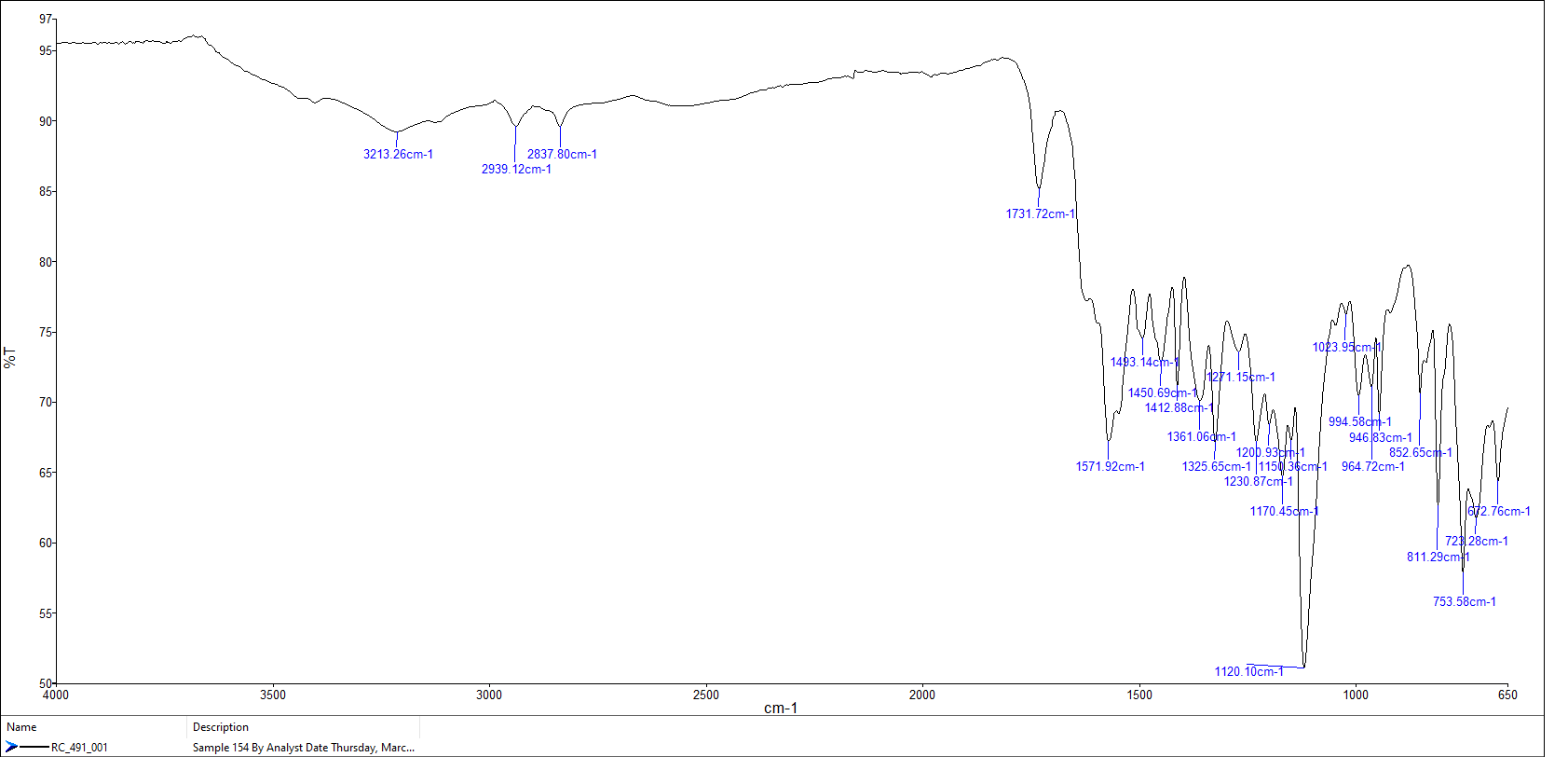
**

Figure S38: FTIR Spectrum for compound **6k**

Figure S39: ^1^H NMR Spectrum for compound **5m**

Figure S40: FTIR Spectrum for compound **5m**

Figure S41: ^1^H NMR Spectrum for compound **6m**

**
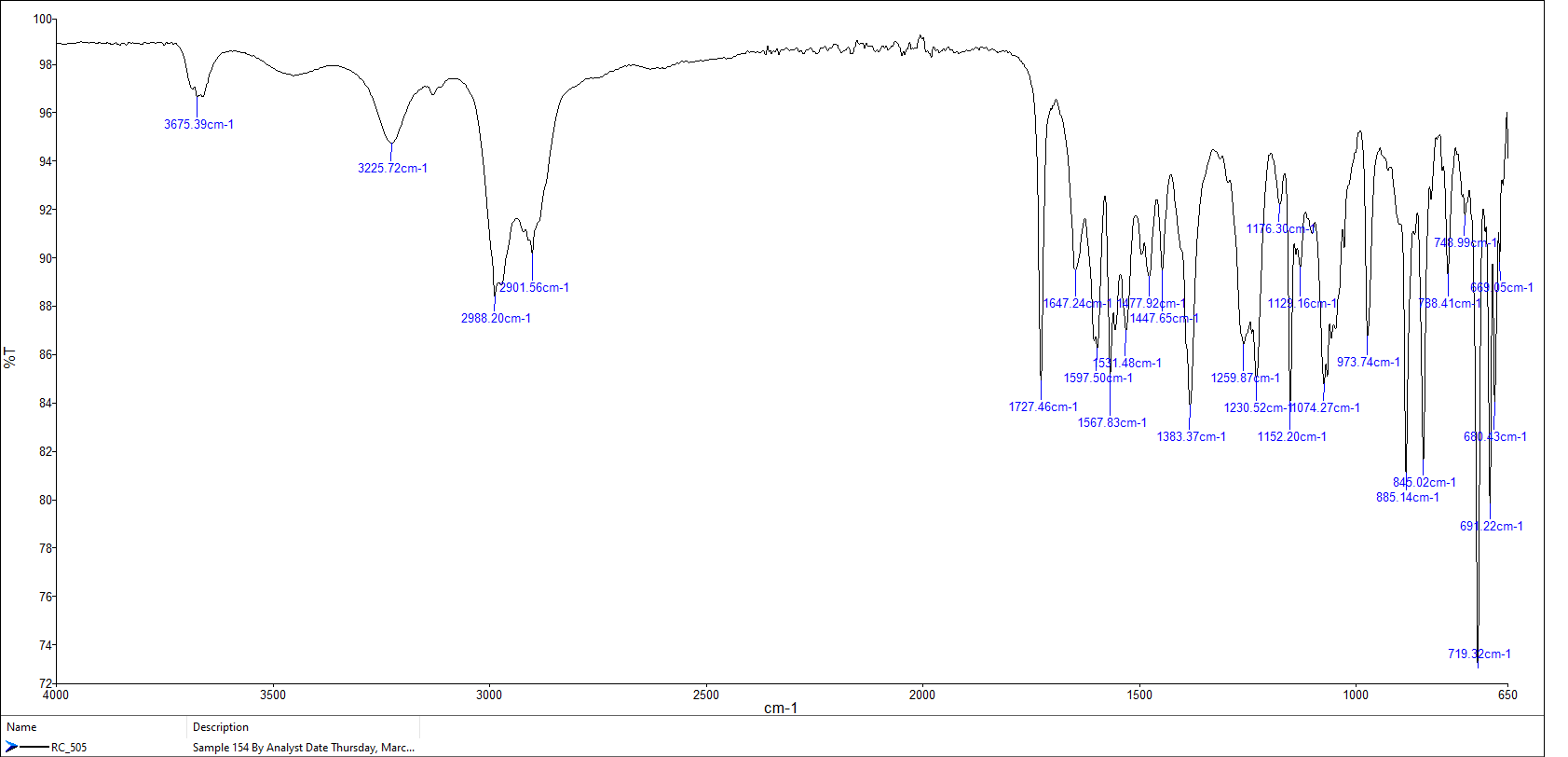
**

Figure S42: FTIR Spectrum for compound **6m**

Figure S43: ^1^H NMR Spectrum for compound **7c**

**
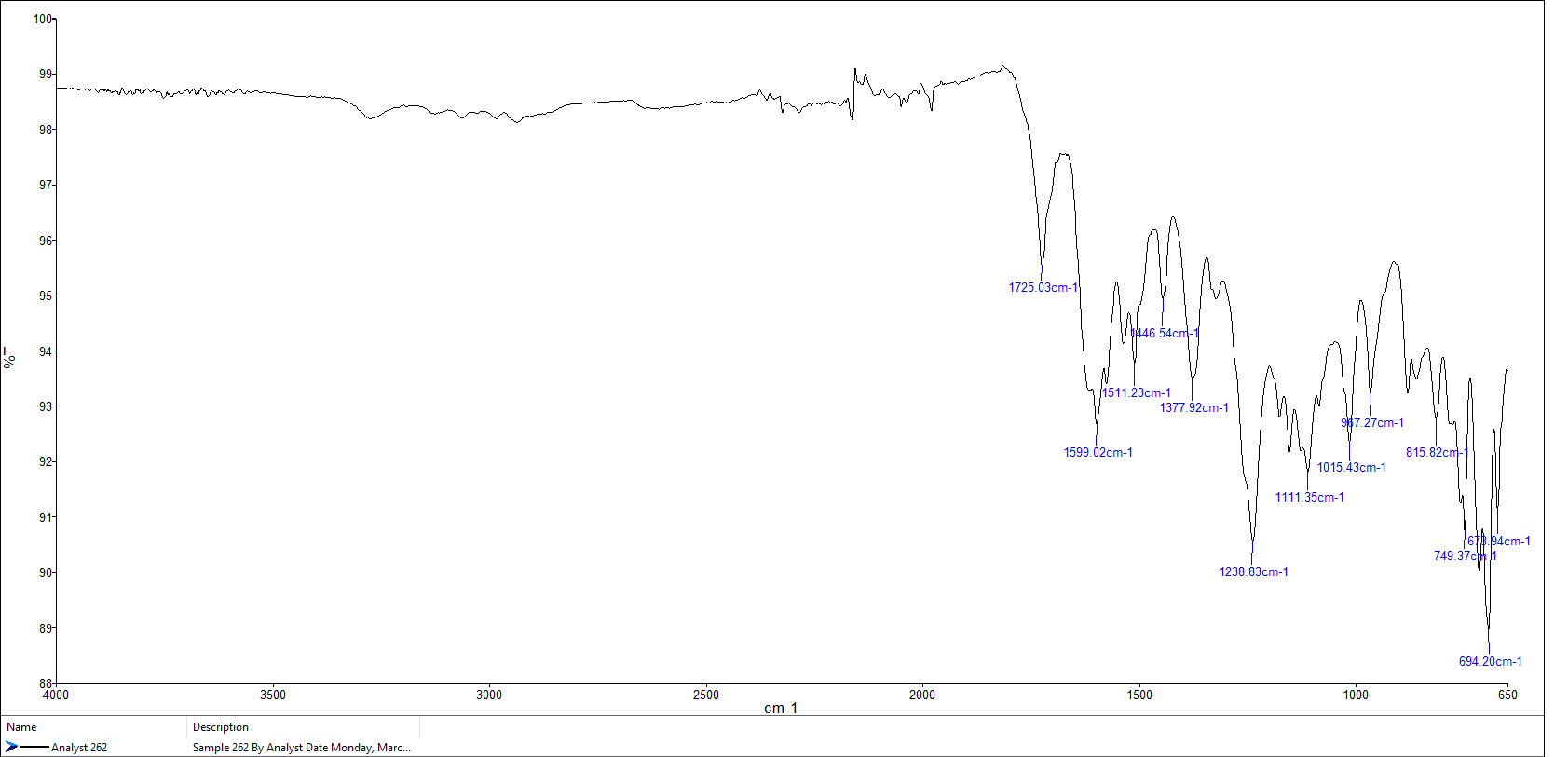
**

Figure S44: FTIR Spectrum for compound **7c**

Figure S45: ^1^H NMR Spectrum for compound **8c**

**
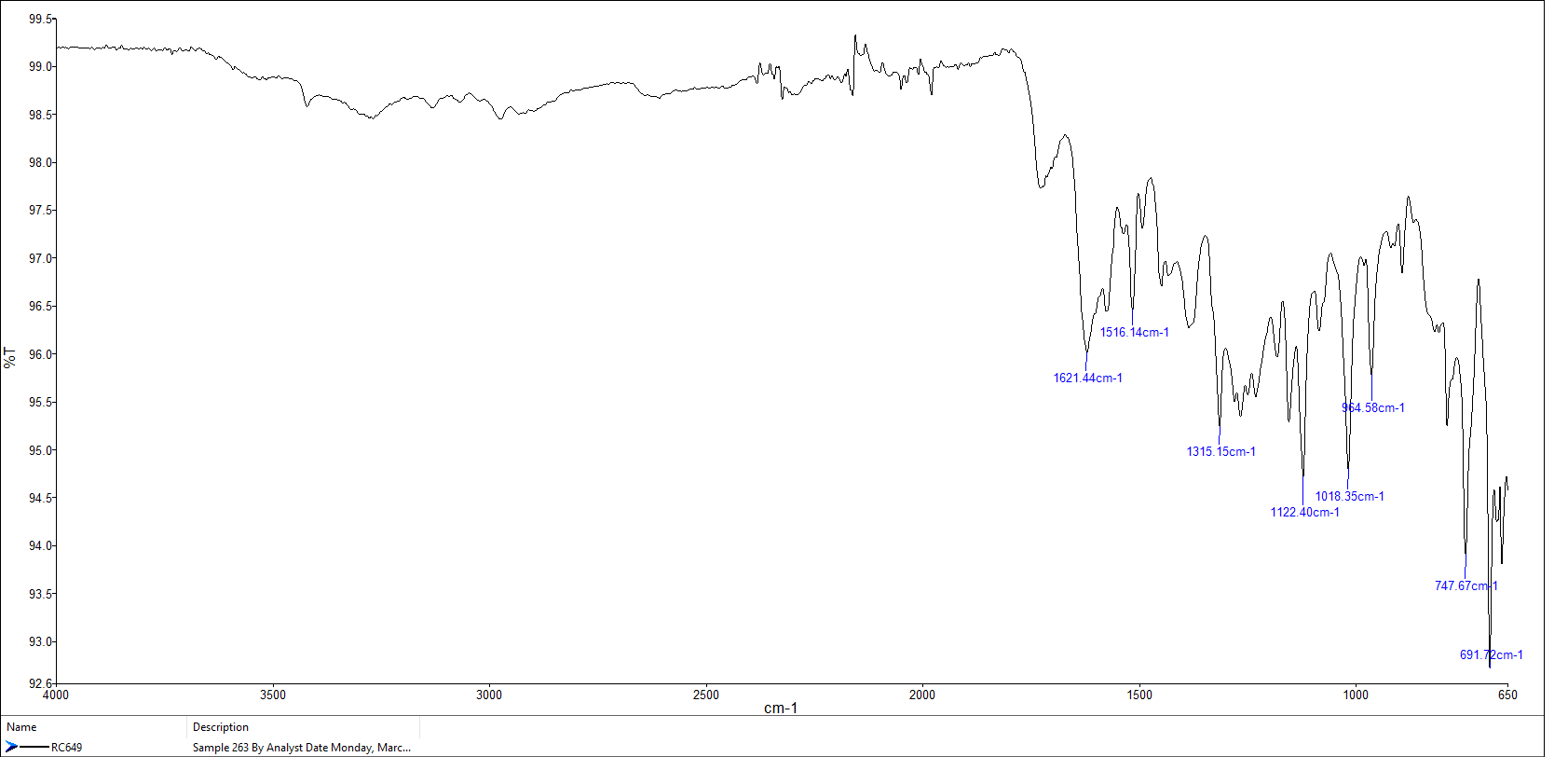
**

Figure S46: FTIR Spectrum for compound **8c**

Figure S47: ^1^H NMR Spectrum for compound **7d**


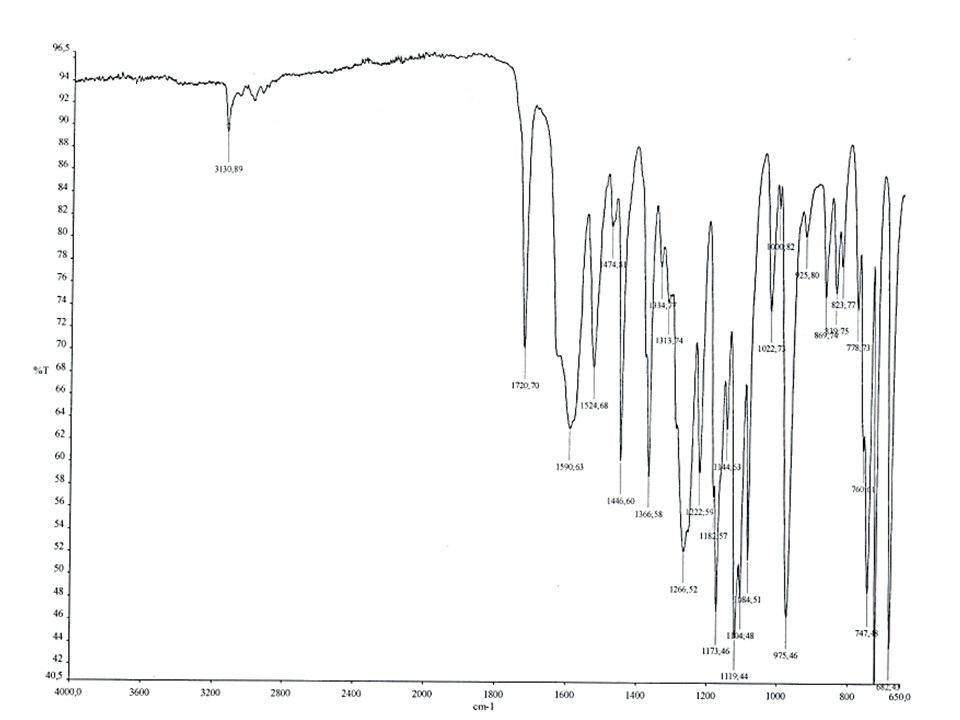


Figure S48: FTIR Spectrum for compound **7d**

Figure S49: ^1^H NMR Spectrum for compound **8d**


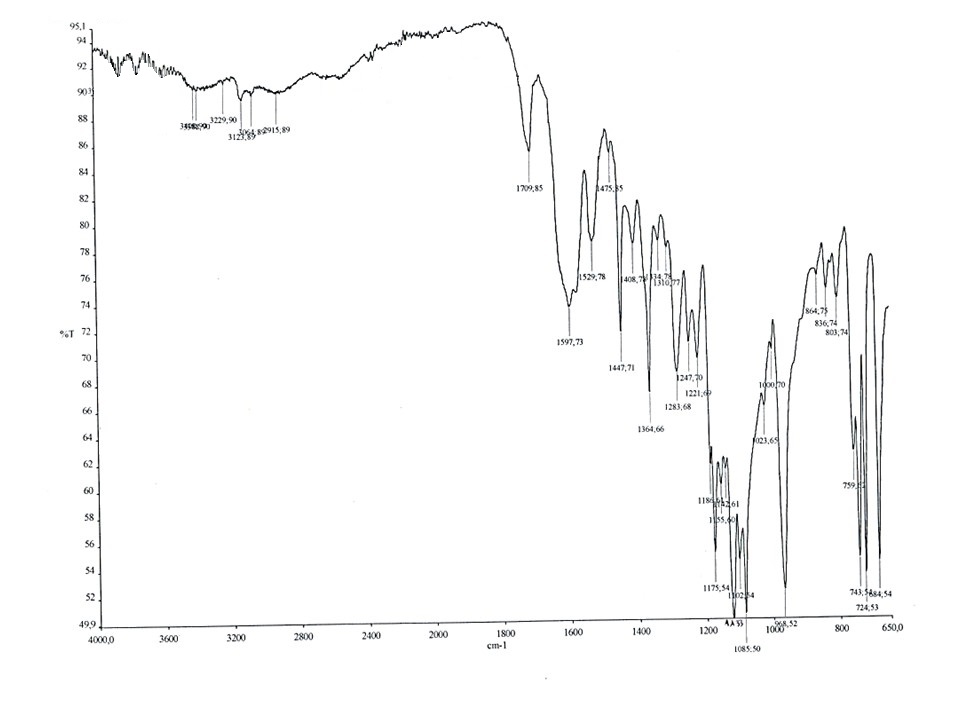


Figure S50: FTIR Spectrum for compound **8d**
