# Supplementary material for: Exploring structure–activity relationships of pyrrolyl diketo acid derivatives as non-nucleoside inhibitors of terminal deoxynucleotidyl transferase enzyme
Source: J Enzyme Inhib Med Chem. 2025 Jun 9;40(1):2496782. doi: 10.1080/14756366.2025.2496782 (PMC12150604; doi:10.1080/14756366.2025.2496782)
Supplement: Table S1.docx [file IENZ_A_2496782_SM8240.docx]

**Table S1.** Pol λ and pol β residual activity after treatment 100 mM of diketo hexenoic derivatives **5a**-**g,j-m**, **6a**-**g,j-m**, **7a**-**d**, **8a**-**d** and diketo butanoic derivatives **9a**,**b**, **10a**,**b**, **11a**,**b** and **12a**,**b**.

| **Cpd** | **pol λ (Pol) Residual activity (%)*^a^*** | | **pol λ (TdT) Residual activity (%)*^b^*** | **pol** β **Residual activity (%)*^c^*** | |
| --- | --- | --- | --- | --- | --- |
|  | **Mg^2+^** | **Mn^2+^** | **Mn^2+^** | **Mg^2+^** | **Mn^2+^** |
| **5a** | 100% | 100% | nd*^d^* | 53% | 100% |
| **5b** | 95% | 100% | 45% | 88% | 95% |
| **5c** | 100% | 100% | 100% | nd | nd |
| **5d** | 27.1% | 70.3% | nd | 10% | 42.7% |
| **5e** | nd | nd | nd | nd | nd |
| **5f** | 60.5% | 75.3% | nd | 5.1% | 33.3% |
| **5g** | 71.3% | 100% | nd | 10% | 100% |
| **5j** | 32.6% | 100% | nd | 23.7% | 100% |
| **5k** | 60.7% | 63.9% | 50.4% | 68% | 100% |
| **5l** | 75.2% | 100% | nd | 52% | 100% |
| **5m** | 20.4% | 64.7% | 100% | 29% | 84.9% |
| **6a** | 90.0% | 77% | 29.6% | 35% | 50.2% |
| **6b** | 84.0% | 98.0% | 50.0% | 81.9% | 92% |
| **6c** | 100% | 100% | 100% | nd | nd |
| **6d** | 33.1% | 100% | nd | 4.44% | 62% |
| **6e** | nd | nd | nd | nd | nd |
| **6f** | 17.9% | 52.1% | nd | 1.88% | 22% |
| **6g** | 89.0% | 100% | nd | 4.14% | 100% |
| **6j** | 15.1% | 100% | nd | 27.1% | 100% |
| **6k** | 37.7% | 20.6% | 42.5% | 73% | 100% |
| **6l** | 40.5% | 100% | nd | 40.1% | 100% |
| **6m** | 11.4% | 21.3% | 50.0% | 45.5% | 62.7% |
| **7a** | 96.0% | 100% | 77% | 82.4% | 95% |
| **7b** | 100% | 100% | 100% | 100% | 69.7% |
| **7c** | 42.3% | 100% | nd | 57% | 100% |
| **7d** | 44.6% | 100% | nd | 67.5% | 100% |
| **8a** | 93.0% | 100% | 59% | 84% | 95% |
| **8b** | 54.6% | 90.7% | 30% | 70.7% | 100% |
| **8c** | 15.6% | 50.7% | nd | 70% | 100% |
| **8d** | 43.2% | 100% | nd | 54.6% | 100% |
| **9a** | 69.0% | 82.3% | 100% | 84% | 100% |
| **9b** | 100% | 100% | 30.1% | 100% | 100% |
| **10a** | 100% | 100% | 57.7% | 86% | 67% |
| **10b** | 93% | 100% | 81.0% | 39.7% | 68.3% |
| **11a** | 100% | 100% | 100% | 94% | 98% |
| **11b** | 70.0% | 94.0% | nd | 60.5% | 88% |
| **12a** | 100% | 100% | 30.1% | 100% | 100% |
| **12b** | 74.0% | 79.0% | 100% | 51.7% | 82.5% |

*^a^*Percentage of pol **λ** polymerase residual activity (%) in presence of Mg^2^**^+^** or Mn^2+^. *^b^*Percentage of pol **λ** TdT residual activity (%) in presence of Mn^2+^. *^c^*Percentage of pol β residual activity (%) in presence of Mn^2+^. *^d^*nd: not determined.
